# Modelling a pathological *GSX2* variant that selectively alters DNA binding reveals hypomorphic mouse brain defects

**DOI:** 10.1242/dmm.052110

**Published:** 2025-02-20

**Authors:** Laura Tweedie, Matthew R. Riccetti, Brittany Cain, Shenyue Qin, Joseph Salomone, Jordan A. Webb, Amy Riesenberg, Lisa A. Ehrman, Ronald R. Waclaw, Rhett A. Kovall, Brian Gebelein, Kenneth Campbell

**Affiliations:** ^1^ Divisions of Developmental Biology, Cincinnati Children's Hospital Medical Center, 3333 Burnet Avenue, Cincinnati, OH 45229, USA; ^2^ Medical Scientist Training Program, University of Cincinnati College of Medicine, Cincinnati, OH 45229, USA; ^3^ Department of Molecular and Cellular Biosciences, University of Cincinnati College of Medicine, Cincinnati, OH 45229, USA; ^4^ Experimental Hematology and Cancer Biology, Cincinnati Children's Hospital Medical Center, 3333 Burnet Avenue, Cincinnati, OH 45229, USA; ^5^ Department of Pediatrics, University of Cincinnati College of Medicine, Cincinnati, OH 45229, USA

**Keywords:** Basal ganglia, Homeodomain, Nucleus tractus solitarius, Striatum, Transcription factor

## Abstract

Gsx2 is a homeodomain transcription factor critical for development of the ventral telencephalon and hindbrain in mouse. Loss of *Gsx2* function results in severe basal ganglia dysgenesis and defects in the nucleus tractus solitarius (nTS) of the hindbrain, together with respiratory failure at birth. De Mori et al. (2019) reported two patients with severe dystonia and basal ganglia dysgenesis that encode distinct recessive *GSX2* variants, including a missense variant within the homeodomain (*GSX2^Q251R^*). Hence, we modelled the homologous *Gsx2* mutation (i.e. *Gsx2^Q252R^*) in mouse, and our biochemical analysis revealed that this variant selectively altered DNA binding. Moreover, mice carrying the *Gsx2^Q252R^* allele exhibited basal ganglia dysgenesis, albeit to a lesser extent than did *Gsx2* null mice. A notable difference between *Gsx2^Q252R^* and *Gsx2* null mice was that *Gsx2^Q252R^* mice survived, and hindbrain analysis revealed relative sparing of the glutamatergic nTS neurons and catecholaminergic A1/C1 and A2/C2 groups. Thus, the *Gsx2^Q252R^* variant is a hypomorph that compromises a subset of Gsx2-dependent neuronal subtypes and highlights a critical role for distinct thresholds of catecholaminergic and/or glutamatergic nTS neurons for viability.

## Teaser

**Summary:** A pathological *GSX2* variant in a critical homeodomain residue selectively reduces DNA binding, and modelling this variant in mouse reveals brain phenotypes similar to those of *Gsx2* null mice, with the exception of postnatal viability.

## INTRODUCTION

The basal ganglia comprise a set of subcortical nuclei that control voluntary movement, motor learning, and aspects of emotion and cognition (Obeso et al., 2014, 2002). Dysfunction within these circuits is found in several developmental disorders, such as dystonia and Tourette's syndrome (Obeso et al., 2014). The striatum (or caudate–putamen) represents the major component of the basal ganglia and derives from progenitors of the medial (MGE) and lateral (LGE) ganglionic eminences within the developing ventral telencephalon (Rubenstein and Campbell, 2020). The LGE gives rise to striatal projection neurons (SPNs), which are GABAergic and send axons to either the globus pallidus via the indirect pathway (iSPNs) or substantia nigra via the direct pathway (dSPNs), while the MGE provides striatal interneurons. The homeodomain (HD) transcription factor (TF) Gsx2 is critical for the correct dorsoventral (DV) patterning and neuronal specification of the LGE progenitors. It does this by upregulating downstream TFs critical for normal striatal development, such as Ascl1 and Dlx factors, and by repressing the expression of factors that specify the dorsal telencephalon, especially Pax6 (Corbin et al., 2000; Toresson et al., 2000; Toresson and Campbell, 2001; Waclaw et al., 2009; Yun et al., 2003, 2001). Accordingly, loss of *Gsx2* expression results in a truncated LGE, leading to a decrease in SPN numbers and striatal size at late embryonic stages. Thus far, postnatal *Gsx2* loss-of-function analysis has been limited (Waclaw et al., 2010) because *Gsx2* null mice are not viable after birth (Szucsik et al., 1997).

Clinical genetic studies recently identified three pathological *GSX2* variants (De Mori et al., 2019; Ürel-Demir et al., 2024). Two of these patients were described in 2019: a 5-year-old female with a nonsense mutation resulting in a severely truncated protein [c.27G>A; p.(S9*)] and a 14-year-old female with a missense mutation in the HD region [c.752A>G; p.(Q251R)] (De Mori et al., 2019). A third female patient was recently described, who died at 2 years of age and had another HD missense mutation [c.747G>C; p.(W249C)] (Ürel-Demir et al., 2024). In the same report, this patient's older sister, who died at 4 months of age, was described as having similar symptoms and magnetic resonance imaging (MRI) findings. Given that the parents were both heterozygous for the *GSX2^W249C^* variant, the authors argued that she was likely affected by the same mutation, although genomic sequencing was not performed. All four patients present remarkably consistent MRI findings, including hypoplasia of the caudate–putamen (i.e. striatum), fusion of the hypothalamus and midbrain, and ventriculomegaly. In addition, the clinical signature in the three confirmed *GSX2* patients was consistent: all patients demonstrated severe dystonia as well as swallowing and feeding difficulties. Notably, both patients described by Ürel-Demir et al. (2024) died owing to respiratory failure.

The nature of the clinical presentations and brain imaging seen in these patients is largely congruent with data from *Gsx2* null mice (Corbin et al., 2000; Toresson et al., 2000; Toresson and Campbell, 2001; Yun et al., 2003, 2001). However, the *GSX2^Q251R^* variant, which potentially generates a defective protein with altered DNA binding characteristics, presents an opportunity to generate a novel mouse model (i.e. *Gsx2^Q252R^*) and gain a deeper understanding of how this variant impacts brain development.

## RESULTS

### Altered DNA binding of the mouse Gsx2^Q252R^ protein

**Fig. 1.**
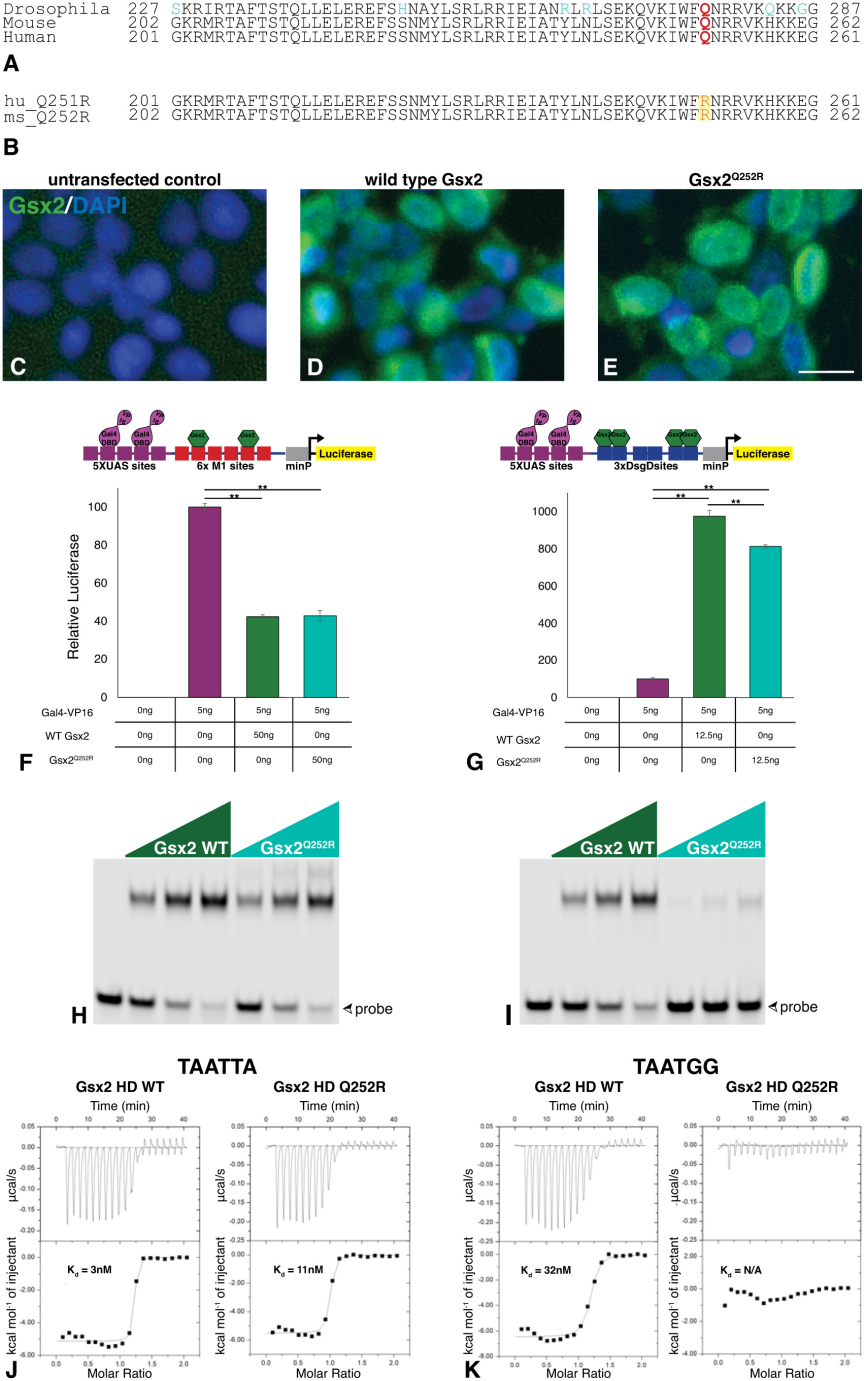
**Gsx2^Q252R^ protein is nuclear localized and transcriptionally active but demonstrates altered DNA binding specificity compared to wild-type (WT) Gsx2**. (A) Amino acid alignment of the Gsx2 homeodomain showing conservation among species, with non-conserved residues in blue and the mutated glutamine (Q252R) in red. (B) Owing to one additional amino acid prior to the homeodomain in the mouse protein, human (hu) R251 is analogous to mouse (ms) R252 (orange text, highlighted in yellow). (C-E) Both Gsx2^Q252R^ and WT Gsx2 are localized to the nucleus in transfected HEK293T cells. DAPI, 4′,6-diamidino-2-phenylindole. Scale bars: 10 μm (with scale bar in E also applicable for C and D). (F,G) In luciferase assays, Gsx2^Q252R^ retained the functions of transcriptional repression (F) and activation (G) at the appropriate DNA sites (high-affinity monomer and dimer sites, respectively; site sequences taken from Salomone et al., 2021). (H,I) Electrophoretic mobility shift assays (EMSAs) were used to test binding to the preferred affinity probe (TAATTA) and the Q50-specific probe (TAATGG). Although both appear to bind TAATTA well, Gsx2^Q252R^ protein binding is substantially reduced on the TAATGG (Q50) probe. (J,K) Isothermal titration calorimetry indicates that both WT Gsx2 and Gsx2^Q252R^ bind the preferred binding site (TAATTA) at high affinities (3 nM for WT Gsx2, 11 nM for Gsx2^Q252R^) (J); however, although the WT Gsx2 binds the Q50-specific site (TAATGG) with a dissociation constant (K_d_) of 32 nM, the Gsx2^Q252R^ protein lacks binding at this site (K). N/A, not applicable.

The Gsx2 HD is highly conserved between orthologues – including *Drosophila* Ind, mouse Gsx2 and human GSX2 – with the latter two being identical (Fig. 1A). All three species possess a glutamine (Q) at position 50 of the HD, which is the altered amino acid in the reported GSX2^Q251R^ variant (De Mori et al., 2019). Mouse Gsx2 has one additional amino acid preceding the HD, and thus the analogous mutation is Gsx2^Q252R^ (Fig. 1B). We will utilize this nomenclature to describe the mouse allele for the remainder of this study. We first set out to characterize the Gsx2^Q252R^ protein *in vitro* to determine how the mutation affects DNA binding and nuclear localization prior to modelling the mutation *in vivo.*

Given the similarly severe basal ganglia dysgenesis observed in the patient with the *GSX2^Q251R^* variant and the patient with the nonsense GSX2^S9*^ null allele, it seemed possible that the missense variant lacked nuclear localization and/or was not transcriptionally active. In fact, De Mori et al. (2019) reported significantly reduced nuclear localization of GSX2^Q251R^ in transfected HeLa cells. We similarly tested the Gsx2^Q252R^ protein in HEK293T cells transfected with either wild-type mouse *Gsx2* or *Gsx2^Q252R^*. Using an antibody against the C-terminus of Gsx2 (Toresson et al., 2000), we found that both proteins localized almost exclusively to the nucleus (Fig. 1C,E). Furthermore, to determine whether the Gsx2^Q252R^ protein is capable of regulating transcription, we made use of our recently published luciferase assays showing that Gsx2 can mediate repression via consensus monomer (M)-sites (TAATTA) or activation via cooperative homodimer (D)-sites (ATTAGAATTTTATTA) (Salomone et al., 2021). Similarly to wild-type Gsx2, the Gsx2^Q252R^ protein can mediate both repression (Fig. 1F) and activation (Fig. 1G) on these high-affinity sites in this cell-based assay.

The Q>R change occurs at the 50th position of the Gsx2 HD, and prior studies showed that residue differences at the 50th position can impact DNA binding specificity (Berger et al., 2008; Bürglin and Affolter, 2016; Laughon, 1991). In particular, Q50 HDs, like Gsx2, are well known to bind TAATTA consensus sites as well as the Q50 consensus TAATGG sequence with high affinity (Webb et al., 2024). To assess whether the Gsx2^Q252R^ variant can similarly bind each of these sequences, we first utilized electrophoretic mobility shift assays (EMSAs) using equimolar concentrations of each protein and labelled TAATTA and TAATGG probes. As expected, wild-type Gsx2 bound both the TAATTA (Fig. 1H) and Q50 HD (TAATGG) (Fig. 1I) probes well, whereas the Gsx2^Q252R^ HD protein bound well to TAATTA (Fig. 1H) but showed considerably reduced binding to the Q50 probe (Fig. 1I). To confirm this loss/reduction of binding to a Q50 consensus sequence, we used isothermal titration calorimetry (ITC) to test DNA binding affinity of the Gsx2^Q252R^ HD protein. In line with the results of Webb et al. (2024), the wild-type Gsx2 HD bound the TAATTA and TAATGG sequences (Fig. 1J,K; Table S1) with low nM affinities, i.e. 3 nM and 32 nM, respectively. Conversely, the Gsx2^Q252R^ protein bound the TAATTA with high affinity (11 nM) but failed to bind the TAATGG Q50 probe (Fig. 1J,K; Table S1). These data suggest that the Gsx2^Q252R^ protein selectively disrupts DNA binding to Q50 HD binding sites (e.g. TAATGG) but not to its consensus TAATTA site. Intriguingly, we examined our published Gsx2 CUT&RUN genomic DNA binding data from the mouse LGE [Gene Expression Omnibus (GEO) GSE162589; Salomone et al., 2021] to determine the number of footprint-confirmed TAATTA and TAATGG (Q50 consensus) sequences within the 2551 called Gsx2 peaks. In total, we found that wild-type Gsx2 binds to 552 TAATTA sites and 277 Q50 sites [out of a total of 5591 footprinted monomer (M)-sites], suggesting that the Gsx2^Q252R^ protein likely exhibits reduced/absent binding to a number of wild-type Gsx2 target sites *in vivo*.

### The Gsx2^Q252R^ protein contributes to hypomorphic basal ganglia phenotypes in the embryonic mouse

**Fig. 2.**
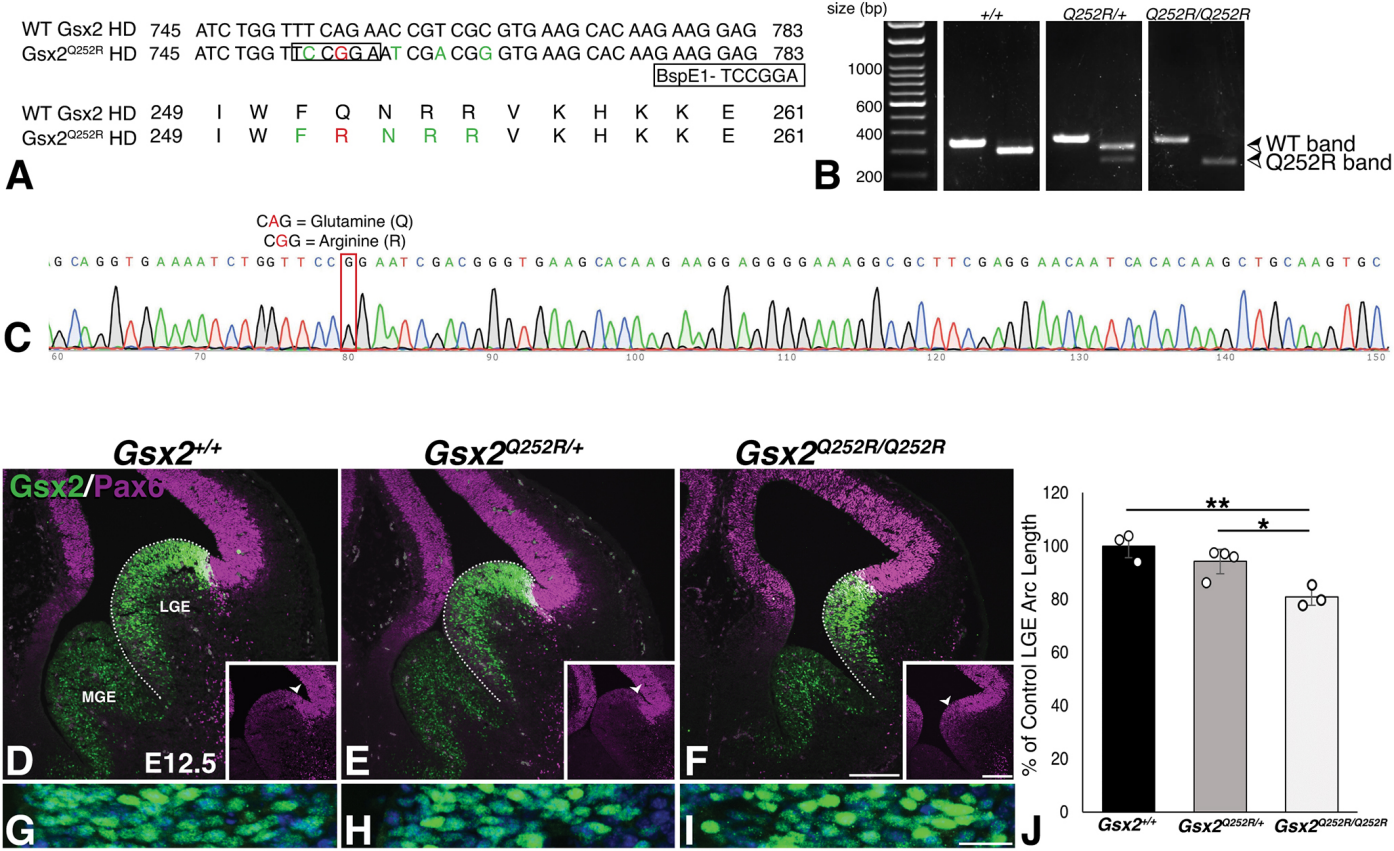
**Gsx2^Q252R^ protein is nuclear localized *in vivo*, and *Gsx2^Q252R/Q252R^*, but not *Gsx2^Q252R/+^*, embryos demonstrate a dorsoventral (DV) patterning defect and lateral ganglionic eminence (LGE) truncation.** (A) Alignment of nucleotide and protein sequences between WT and Gsx2^Q252R^. The Q252R mutation is highlighted in red; green residues indicate silent mutations included for genotyping purposes. A BspE1 restriction enzyme site was generated in the mutant (shown as boxed nucleotides in A), allowing allele-specific digestion in mice carrying the Q252R mutation. (B) Genotyping gel showing three animals from left to right: *Gsx2^+/+^*, *Gsx2^Q252R/+^* and *Gsx2^Q252R/Q252R^*. PCR generates a 348 bp product, and subsequent BspE1 digest yields a 242 bp product for the mutant allele. (C) Sanger sequencing of a homozygous Q252R mutant confirms the expected A to G mutation (red box). (D-F) As seen in previous *Gsx2* mutants, *Gsx2^Q252R/Q252R^* mice demonstrate truncation of the Gsx2 expression domain and resulting Pax6 expansion ventrally (arrowheads in insets indicate this Pax6 expansion). E, embryonic day; MGE, medial ganglionic eminence. (G-I) Gsx2^Q252R^ protein is nuclear localized *in vivo*. (J) Quantification of the arc of the Gsx2 expression domain within the LGE (indicated by the dotted white lines in D-F) indicates a decrease in *Gsx2^Q252R/Q252R^* embryos (80.9±3.3%) compared to that in WT embryos (100±2.5%), and no significant difference between WT and *Gsx2^Q252R/+^* embryos (94.2±2.4%). Bar graphs show average cell count±s.e.m., with individual points representing each embryo. One-way ANOVA with Tukey post hoc was used to determine significance (*n*=3, *P<0.05, **P<0.01). Scale bars: 200 µm (F, inset), 50 µm (I).

To assess the impact of the human *GSX2^Q251R^* HD variant on the development of the mouse brain, we used CRISPR-Cas9 and a donor oligonucleotide approach to change the codon encoding the ‘Q’ (CAG) to ‘R’ (CGG), i.e. c.755A>G; p.Q252R. This mutation thereby models the missense HD variant (c.752A>G; p.Q251R) described in De Mori et al. (2019) (Fig. 2A-C). Not only were the *Gsx2^Q252R/+^* mice viable and fertile, but, unlike *Gsx2* homozygous null mice, which die at birth (Szucsik et al., 1997), *Gsx2^Q252R/Q252R^* mice were viable and fertile.

To generate *Gsx2^Q252R/Q252R^* embryos for analysis, we crossed male and female *Gsx2^Q252R/+^* mice and collected embryos at embryonic day (E)12.5 to assess the impact on telencephalic development. Confirming our *in vitro* results, the Gsx2^Q252R^ protein was indeed localized to the nucleus in LGE progenitors *in vivo* (Fig. 2F,I), similar to wild-type Gsx2 protein in control mice (Fig. 2D,G). At E12.5, wild-type Gsx2 was expressed in progenitors throughout the ventricular zone (VZ) of the LGE and MGE, with a high-dorsal to low-ventral gradient in the LGE. Conversely, Pax6 was expressed at high levels in progenitors throughout the pallial VZ and met Gsx2-expressing LGE progenitors at the pallio-subpallial boundary (Fig. 2D). Gsx2 is known to play a critical role in DV patterning of the LGE by repressing dorsal transcriptional programs directed, in part, by the paired HD TF Pax6 (Corbin et al., 2000; Toresson et al., 2000; Toresson and Campbell, 2001; Waclaw et al., 2009; Yun et al., 2001, 2003). Thus, in the absence of *Gsx2* function, Pax6 is upregulated in LGE progenitors and effectively truncates the *Gsx2* null LGE. Accordingly, we observed a ventral shift in Pax6 expression in the *Gsx2^Q252R/Q252R^* LGE and a concomitant reduction in the Gsx2 expression domain (Fig. 2F) compared to that in wild-type (Fig. 2D) and *Gsx2^Q252R/+^* (Fig. 2E) embryos. We quantified the truncation of the Gsx2-expressing domain in the *Gsx2^Q252R/Q252R^* LGE and found a 19% reduction, which was significant in both wild-type and heterozygous embryos (Fig. 2J). Therefore, the *Gsx2^Q252R^* allele at least partially phenocopies the *Gsx2* null when in a homozygous state, similar to the *GSX2^Q251R/Q251R^* patient reported by De Mori et al. (2019).

MRI scans revealed similar basal ganglia dysgenesis in patients with the null and missense HD variants (De Mori et al., 2019). However, because only one patient with each variant was available for analysis, a rigorous comparison of each phenotype was not possible. The generation of the *Gsx2^Q252R^* allele in mice allowed us to perform robust comparisons with the *Gsx2* null as well as assess whether there were any changes unique to the *Gsx2^Q252R^* mice. For these comparisons, we utilized a previously generated enhanced GFP (EGFP) knock-in knockout allele (i.e. *Gsx2^EGFP^*), in which EGFP is expressed in place of Gsx2 (Wang et al., 2009). This allele allowed us to use EGFP to mark and compare *Gsx2* expression domains even in the absence of Gsx2 protein. Additionally, we utilized another null allele in which the floxed second exon of *Gsx2* has been recombined in the germline (i.e. recombined allele, Gsx2^RA^) (Waclaw et al., 2009). Previous studies have shown no observed phenotypes in *Gsx2^EGFP/+^* or *Gsx2^RA/+^* mice (Waclaw et al., 2009; Wang et al., 2009), making them useful controls for our studies.

**Fig. 3.**
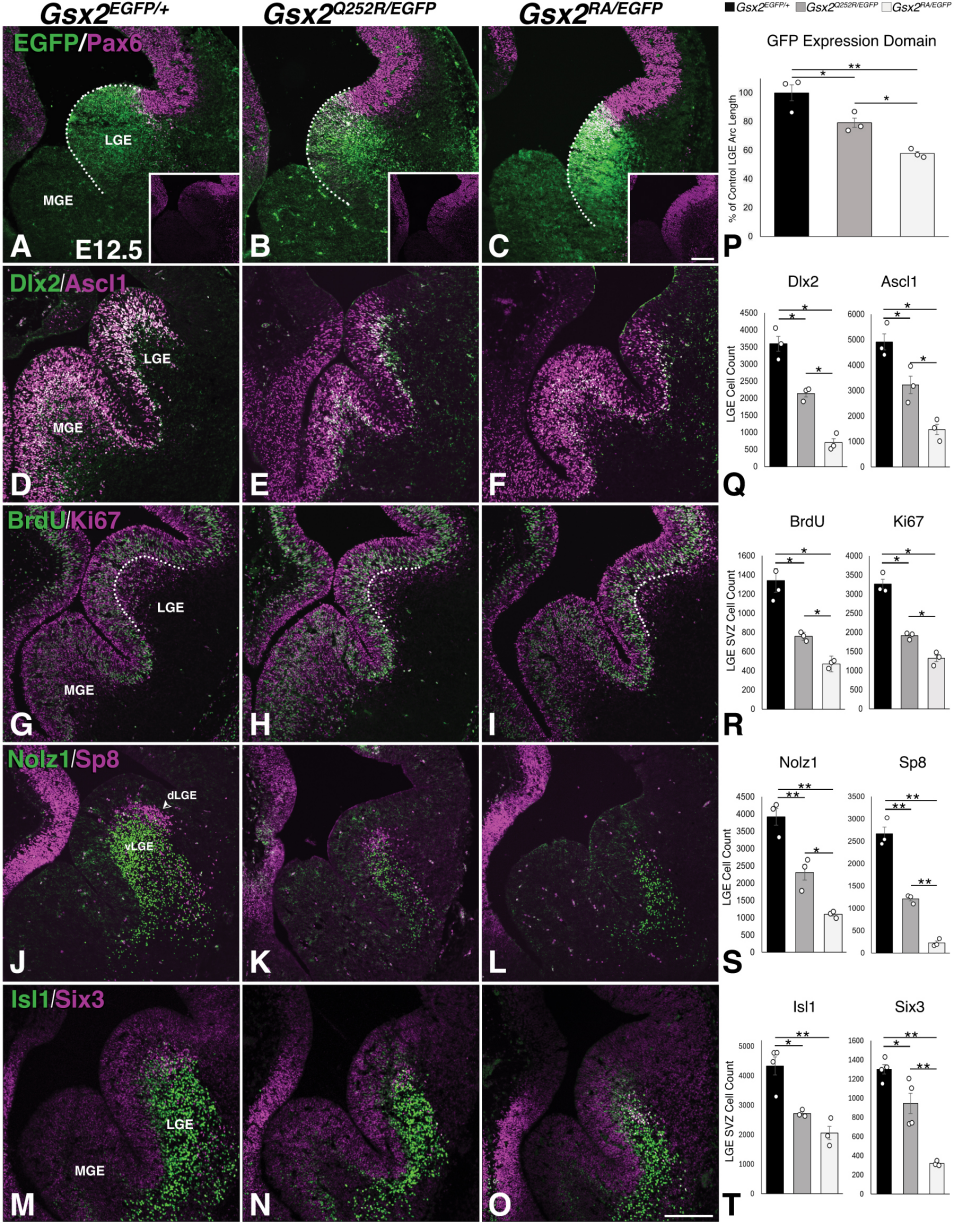
**Intermediate phenotype of *Gsx2^Q252R^* mutants compared to that of *Gsx2* nulls suggests that the Gsx2^Q252R^ protein is a hypomorph.** (A-C) Compared to control embryos (A), *Gsx2^Q252R/EGFP^* embryos demonstrate a truncated EGFP (i.e. *Gsx2*) expression domain within the LGE (B,P), similar in magnitude to that seen in *Gsx2^Q252R/Q252R^* embryos (see Fig. 2J), but less severe than that in *Gsx2* null embryos (C,P). The degree of the LGE truncation correlates with the expansion of Pax6 in the *Gsx2* null versus *Gsx2^Q252R/EGFP^* embryos (see insets in A-C). (D-F) The expression of ventral telencephalic transcription factors Ascl1 and Dlx2 in LGE progenitors is severely truncated in *Gsx2* null embryos (F), and more moderately truncated in *Gsx2^Q252R/EGFP^* embryos (E), compared to that in controls (D). (G-I) Proliferation analysis was performed using a 1 h bromodeoxyuridine (BrdU) pulse and immunofluorescence for Ki67. *Gsx2^Q252R/EGFP^* embryos exhibit intermediate loss of Ki67^+^ LGE subventricular zone (SVZ) cells (H) compared to those in *Gsx2* null (I) and control (G) embryos. Note, similar proportions of Ki67 SVZ cells were in S-phase among the genotypes, indicating no change in cell cycle. (J-L) Markers of dorsal LGE (dLGE, arrowhead) and ventral LGE (vLGE), Sp8 and Nolz1, respectively, are expressed in distinct compartments of the control LGE (J). Again, *Gsx2^Q252R/EGFP^* embryos show an intermediate phenotype (K) compared to that of the *Gsx2* nulls (L). Note the almost complete lack of Sp8 (dLGE) in the null LGE (L). (M-O) As Gsx2 progenitors mature, the resulting SPNs become either direct (Isl1^+^) or indirect (Six3^+^) pathway neurons. In both *Gsx2^Q252R/EGFP^* and *Gsx2* null mice, Isl1 is prematurely expressed in the ventricular zone (VZ), suggesting precocious differentiation of these cells. (P) The arc of EGFP expression within the LGE (indicated by the dotted white lines in A-C) is 79.2±3.3% of the control length in *Gsx2^Q252R/EGFP^* embryos, and 57.7±1.4% of the control length in *Gsx2^RA/EGFP^* embryos. (Q) The decrease in Dlx2^+^ and Ascl1^+^ cells is intermediate within the *Gsx2^Q252R/EGFP^* LGE (2135±94.1 and 3223.7±338.9 cells, respectively) compared to that in the control LGE (3597±217.3 and 4912±314.9 cells, respectively) and the null LGE (704±115.4 and 1472±202.8 cells, respectively). (R) Quantification of Ki67^+^ and BrdU^+^ LGE SVZ cells (located below the dotted white line in G-I) shows a moderate decrease in *Gsx2^Q252R/EGFP^* mice (1915.3±42.6 and 759.7±21.8 cells, respectively), and a severe reduction in *Gsx2* nulls (1322.3±82.9 and 471.7±19.9 cells, respectively), compared to those in controls (3264±124.2 and 1341.5±73.9 cells, respectively). (S) The Nolz1^+^ and Sp8^+^ cell counts in the *Gsx2^Q252R/EGFP^* LGE are 2311.7±221.4 and 1212.7±46.4 cells, respectively, compared to 3919±241.1 and 2670.3±147.4 cells, respectively, in control LGE, and 1097.7±45.4 and 227.7±39.3 cells, respectively, in *Gsx2* null LGE. (T) Quantification of Isl1^+^ and Six3^+^ SVZ cells indicates a significant decrease in both cell types in the LGE in *Gsx2* null embryos (2055.3±222.2 and 319.7±12 cells, respectively), and to a lesser extent in *Gsx2^Q252R/EGFP^* embryos (2721.7±55.4 and 947±106 cells, respectively), compared to those in control embryos (4331.8±306.1 and 1302±49.8 cells, respectively). Bar graphs show average cell count±s.e.m. Significance for quantifications determined using one-way ANOVA with Tukey post hoc (*n*=3 for each genotype, *P<0.05, **P<0.01). Scale bars: 200 µm.

We compared LGE development in E12.5 *Gsx2^Q252R/EGFP^* embryos expressing only the Gsx2^Q252R^ protein with that of *Gsx2^EGFP/RA^* (null) and *Gsx2^EGFP/+^* (control) embryos. As mentioned above, the EGFP expression domain serves as a proxy for *Gsx2* expression, even in null embryos. We measured a 42±1% reduction in the circumference of the EGFP-expressing domain in the LGE of *Gsx2* null (*Gsx2^RA/EGFP^*) embryos compared to that in control embryos (*Gsx2^EGFP/+^*) (Fig. 3A,C,P). In comparison, the EGFP expression domain in the LGE of *Gsx2^Q252R/EGFP^* embryos was 21±3% reduced compared to that in controls (Fig. 3A,B,P), and was significantly different from that in *Gsx2* null embryos (Fig. 3P); this difference was nearly identical to that observed between *Gsx2^Q252R/Q252R^* and wild-type embryos (i.e. 19±3%, see Fig. 2J). Consequently, Pax6 was expanded further ventrally into the *Gsx2* null LGE (Fig. 3C, inset) than in the *Gsx2^Q252R/EGFP^* LGE (Fig. 3B, inset), corresponding to the intermediate truncation observed. As a result, Ascl1 and Dlx2, which are well-known regulators of basal ganglia development (reviewed in Rubenstein and Campbell, 2020), were severely downregulated and truncated in LGE progenitors in E12.5 *Gsx2* null embryos (Fig. 3F,Q) (Corbin et al., 2000; Toresson et al., 2000; Waclaw et al., 2009; Wang et al., 2013, 2009; Yun et al., 2001), whereas E12.5 *Gsx2^Q252R/EGFP^* embryos exhibited an intermediate loss of these TFs in LGE progenitors (compare Fig. 3E with D,F,Q). These data largely reflect the significant, but less severe, Pax6 expansion and LGE truncation in *Gsx2^Q252R/EGFP^* compared to *Gsx2* null mice.

Previous studies (Toresson et al., 2000; Yun et al., 2001) ruled out cell death as a contributing factor to the LGE phenotype in *Gsx2* nulls, and we observed no change in LGE cell death in the *Gsx2^Q252R/EGFP^* embryos (Fig. S2). To assess the effect of the observed LGE truncations on proliferating progenitors, we performed 1 h bromodeoxyuridine (BrdU) pulse-chase experiments and Ki67 (also known as Mki67) staining in the two *Gsx2* mutant backgrounds, allowing us to identify progenitors in S-phase and to delineate the proliferative LGE subventricular zone (SVZ). The number of S-phase LGE progenitors was proportionate to the truncations observed in *Gsx2* null (Fig. 3I,R) and *Gsx2^Q252R/EGFP^* (Fig. 3H,R) embryos compared to controls (Fig. 3G). Because our previous study did report reduced Ki67-positive SVZ progenitors within the *Gsx2* null LGE (Toresson and Campbell, 2001), we quantified Ki67 cells in the LGE SVZ of *Gsx2^Q252R/EGFP^* embryos and found numbers intermediate to those of the *Gsx2* null and control embryos (Fig. 3G-I,R). The proportion of S-phase progenitors among the Ki67-labelled LGE SVZ progenitors was not different between the two *Gsx2* mutants and controls (ranging between 33% and 43%), suggesting that LGE SVZ progenitors divide at the same rate regardless of genotype.

LGE progenitors sequentially give rise to three distinct neuronal subtypes: the ventral (v)LGE gives rise to SPNs whereas the dorsal (d)LGE gives rise to the olfactory bulb interneurons and intercalated cells of the amygdala (Kuerbitz et al., 2021, 2018; Stenman et al., 2003; Waclaw et al., 2010, 2006). At E12.5, the vLGE can be marked by Nolz1 (also known as Zfp503) (Ko et al., 2013), whereas the dLGE is labelled by Sp8 (Waclaw et al., 2006) (Fig. 3J). As previously reported (Stenman et al., 2003; Waclaw et al., 2009), the *Gsx2* null LGE shows severe reduction of the vLGE and nearly complete loss of the dLGE (Fig. 3L,S) at E12.5. In the *Gsx2^Q252R/EGFP^* embryos, Nolz1 and Sp8 expression domains exhibit intermediate reduction, in line with the partial LGE truncation observed in these embryos (compare Fig. 3J-L,S). The vLGE gives rise to two subtypes of SPNs, namely the dSPNs and iSPNs, which can be labelled by Isl1 (Ehrman et al., 2013; Lu et al., 2014) and Six3 (Song et al., 2021; Xu et al., 2018), respectively. Already at E12.5, when the earliest SPNs are being generated, Isl1 and Six3 mark distinct populations of LGE SVZ cells and newborn striatal neurons in control embryos (Fig. 3M). We previously showed that the truncation of the LGE in *Gsx2* nulls leads to a severe reduction in Isl1^+^ cells in the developing striatum (Toresson and Campbell, 2001), and this reduction was evident at E12.5 within the LGE SVZ (Fig. 3O,T). At this developmental stage, however, Isl1^+^ SVZ cells in *Gsx2^Q252R/EGFP^* embryos showed a more modest reduction from those in controls (Fig. 3M-O,T). The iSPN marker, Six3, was severely reduced in the *Gsx2* null LGE SVZ region (Fig. 3O), while the number of cells expressing this marker in the same region in *Gsx2^Q252R/EGFP^* embryos was, again, intermediate (Fig. 3M-O,T).

**Fig. 4.**
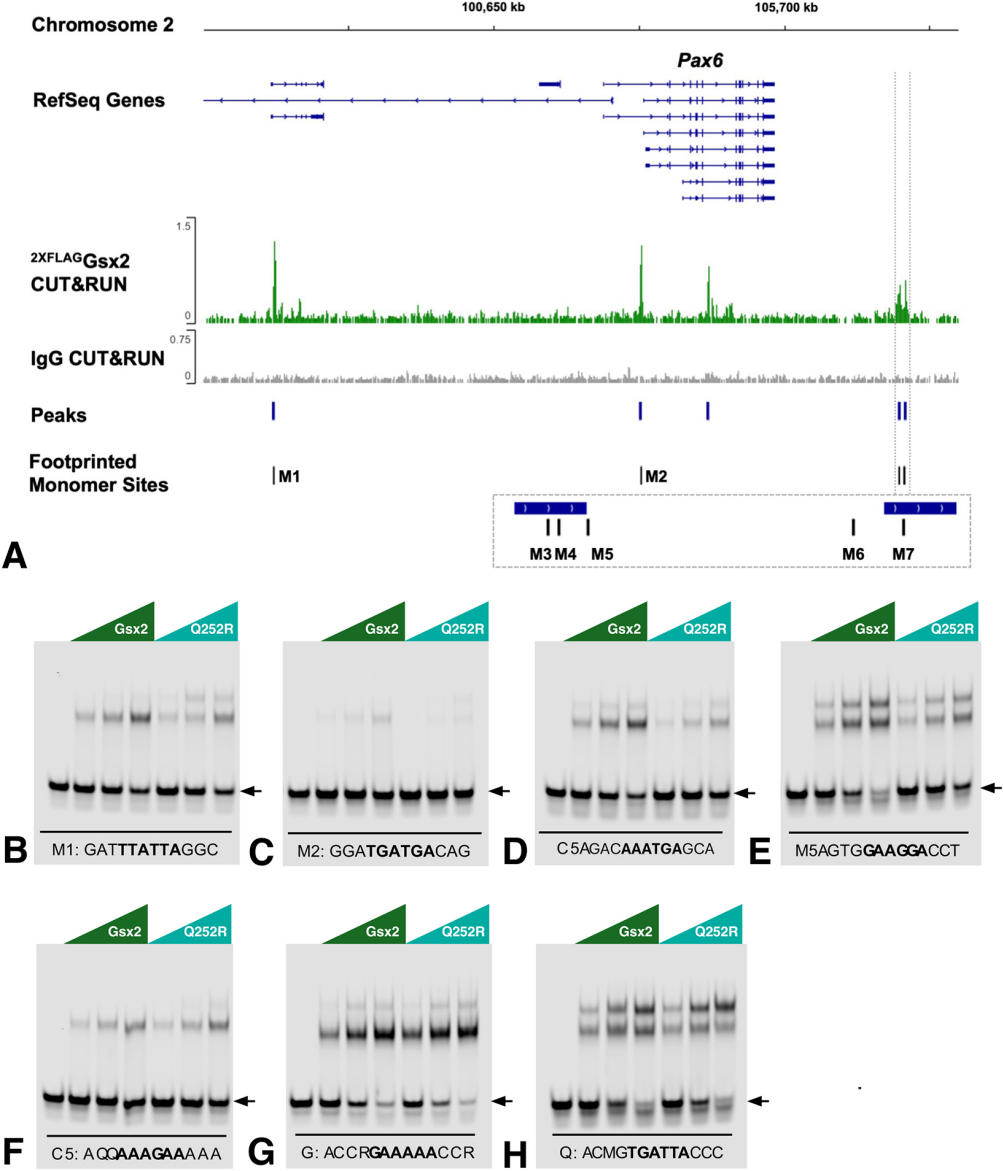
**Reduced binding of Gsx2^Q252R^ to selected footprinted regions around the *Pax6* locus.** (A) Genomic region of the *Pax6* locus showing the locations of Gsx2 CUT&RUN peaks from Salomone et al. (2021) and associated M-site footprinted regions (M1-M7). (B-H) EMSAs were performed using equimolar concentrations of wild-type Gsx2 and Gsx2^Q252R^ to test binding to DNA probes containing the footprinted regions (M1-M7). Wild-type Gsx2 bound well to M1 (B) and M3-M7 probes (D-H), with weak binding to M2 (C). Conversely, the Gsx2^Q252R^ protein showed reduced binding to M1-M4 probes (B-E), and its binding to M5-M7 probes was nearly identical to that of wild type (F-H). Probes (M1-M7) are indicated by the arrows and by sequences below the gel, with the protected region in bold (B-H).

As mentioned above, ectopic Pax6 in *Gsx2* mutant LGE progenitors contributes to central nervous system DV patterning defects resulting in truncation of the LGE. Accordingly, our prior Gsx2 genomic DNA binding data (GEO GSE162589) identified five CUT&RUN binding peaks with seven footprinted M-sites (M1-M7) near the *Pax6* locus (Salomone et al., 2021) (Fig. 4A). To assess the ability of the Gsx2^Q252R^ variant to bind these sites, we performed comparative DNA binding assays using equimolar concentrations of purified wild-type Gsx2 and Gsx2^Q252R^ proteins. It should be noted that only one of the sequences (M4), contained a consensus (i.e. TAATTA) motif, and none had a perfect match for the Q50 motif (i.e. TAATGG). In fact, together, these two consensus sequences only comprised ∼15% of the 5591 footprinted M-sites previously identified (Salomone et al., 2021), indicating significant degeneracy in wild-type Gsx2 DNA binding sites. Wild-type Gsx2 bound all seven probes but with differing efficacy (Fig. 4B-H). Moderate to strong binding was observed to the M1 and M3-M7 probes with weak binding to the M2 probe. Interestingly, the Gsx2^Q252R^ protein displayed reduced binding to the M1-M3 probes (Fig. 4B-D) and a slight reduction in binding to the M4 probe, containing the TAATTA consensus sequence (Fig. 4E). No obvious difference in binding between the wild-type and mutant protein to the M5-M7 probes was apparent (Fig. 4F-H). Therefore, the difference between wild-type Gsx2 and Gsx2^Q252R^ binding to identified Gsx2 binding sites around the *Pax6* locus could provide a mechanism for the partial Pax6 expansion observed in the *Gsx2^Q252R/Q252R^* and *Gsx2^Q252R/EGFP^* LGE, compared to that in null LGE (see Figs 2 and 3). Taken together, these findings indicate that the Gsx2^Q252R^ protein is less effective at repressing *Pax6* than wild-type Gsx2, leading to the intermediate LGE truncation and misspecification observed in *Gsx2^Q252R/EGFP^* embryos, suggesting that the mutant protein is a hypomorph.

### Expansion of Gsx1 in the *Gsx2^Q252R/EGFP^* LGE

**Fig. 5.**
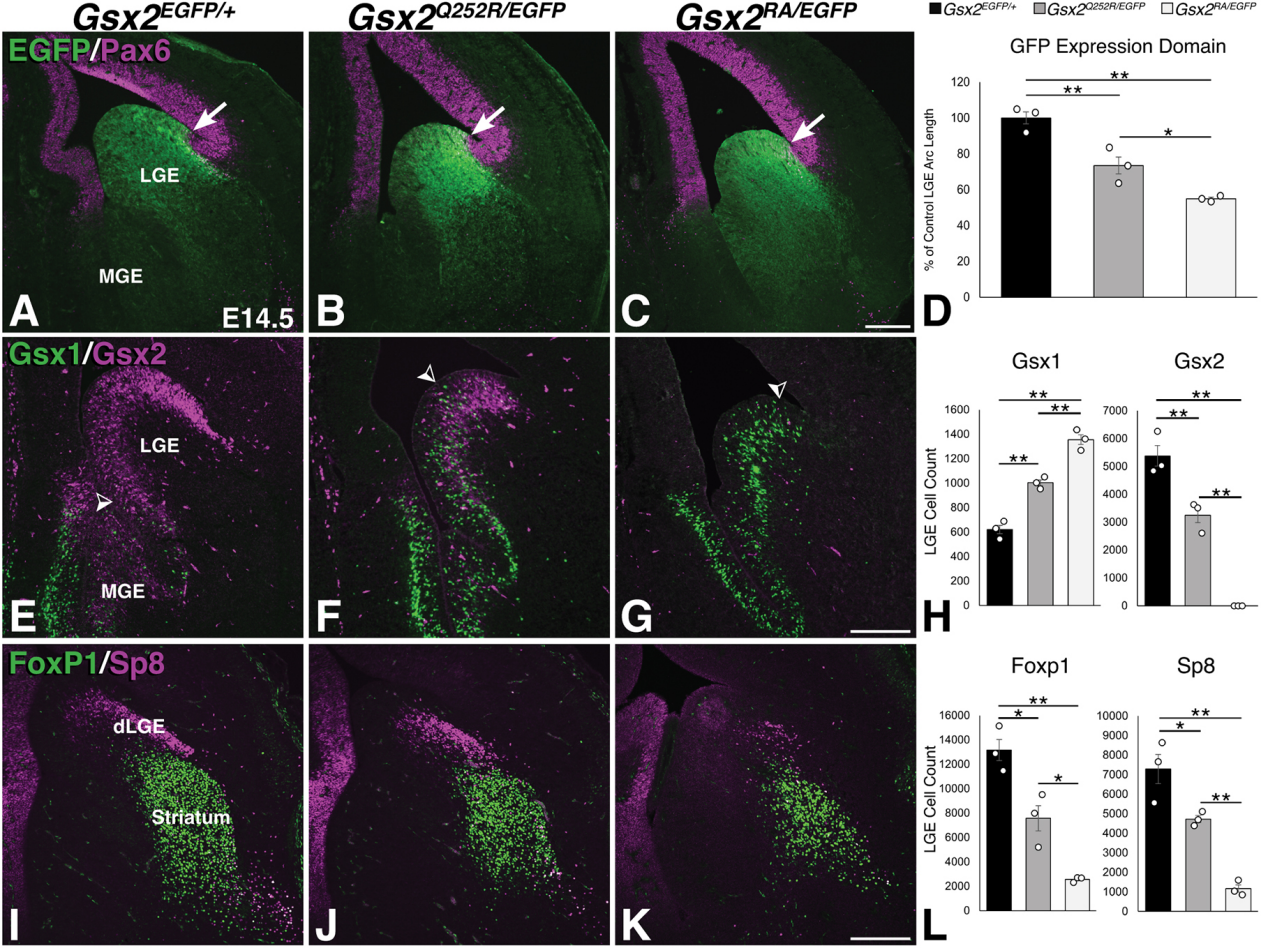
**Molecular recovery in the E14.5 LGE of *Gsx2^Q252R/EGFP^* embryos, showing intermingling of Gsx1- and Gsx2^Q252R^-expressing cells in the dorsal half of the LGE**. (A-C) Immunofluorescence for Pax6 and EGFP (to define the LGE) at E14.5, showing a newly formed pallio-subpallial boundary (arrows in A-C) in both *Gsx2* mutants. (E-G) Gsx1 is restricted to the ventralmost LGE in controls (arrowhead in E) and is moderately upregulated in the *Gsx2^Q252R/EGFP^* LGE (F), showing high expression in the ventral half of the mutant LGE, and intermingling of Gsx1- and Gsx2^Q252R^-expressing cells in the dorsal half (arrowhead in F). In *Gsx2* nulls, Gsx1 is robustly upregulated throughout the DV extent of the LGE (arrowhead) at this stage (G). (I-K) Improved recovery of both LGE compartments: vLGE (Foxp1 marking SPNs) and dLGE (Sp8^+^) in *Gsx2^Q252R/EGFP^* mutants (J) compared to *Gsx2* nulls (K) and controls (I). (D) The EGFP expression arc is 73.5±4.7% of that of the control in *Gsx2^Q252R/EGFP^* embryos and 55±0.8% of that of the control in *Gsx2* null embryos. (H) Quantifications indicate a decrease in Gsx2-expressing cells in *Gsx2^Q252R/EGFP^* mice (3251.3±265.9 cells compared to 5381±364.8 cells in the control), and upregulation of Gsx1-expressing cells in these embryos (1003±23.4 cells compared to 620±34.4 cells in the control). This increased expression is more pronounced in the *Gsx2* null embryos (1355.3±39.9 cells). (L) Quantification of Foxp1 and Sp8 cells also showed an intermediate decrease in *Gsx2^Q252R/EGFP^* embryos (7569.7±1032.2 and 4732±173.3 cells, respectively, compared to 13,170.7±870 and 7287.7±744.8 cells, respectively, in the control), which is not as severe as that in the Gsx2 null embryos (2567.3±106 and 1167.3±184.8 cells, respectively). Significance in quantifications (D,H,L) was determined using one-way ANOVA with Tukey post hoc (*n*=3 for each genotype, *P<0.05, **P>0.01). Quantifications are shown in bar graphs as average cell count±s.e.m. Scale bars: 200 µm.

At later embryonic stages (i.e. E14 and onward), a new pallio-subpallial boundary is established in the *Gsx2* null mutant where Pax6 and the normal Gsx2 expression domain abut (Toresson et al., 2000; Yun et al., 2001). Based on the EGFP expression domain in the *Gsx2^Q252R/EGFP^* LGE, it appears that the pallio-subpallial boundary has been re-established (Fig. 5B); however, the truncated LGE remains intermediate between that of control and *Gsx2* nulls at E14.5 (Fig. 5A,C,D). The Gsx2 family member, Gsx1, is expressed in a largely complementary manner, with high levels in the MGE and the ventralmost vLGE (Fig. 5E). However, in the absence of *Gsx2*, Gsx1 is robustly upregulated throughout the mutant LGE from E14 onward (Toresson and Campbell, 2001; Wang et al., 2009; Yun et al., 2003, 2001). In fact, the limited striatal development observed in *Gsx2* null embryos is critically dependent on *Gsx1* function (Toresson and Campbell, 2001; Yun et al., 2003). Again, in this study, by E14.5, Gsx1 expression was upregulated throughout the DV extent of the LGE in *Gsx2* nulls (Fig. 5G,H) compared to that in controls (Fig. 5E). Interestingly, Gsx1 was also strongly upregulated within the ventral half of the *Gsx2^Q252R/EGFP^* LGE and even expressed in scattered progenitors of the dorsal half intermingled with the majority Gsx2^Q252R^-expressing progenitors (Fig. 5F,H). Thus, the *Gsx2^Q252R/EGFP^* mutant LGE has a mixture of progenitors expressing Gsx2^Q252R^ and Gsx1 proteins, which might more efficiently promote vLGE and dLGE specification than Gsx1 alone in the *Gsx2* nulls. To assess this in the different *Gsx2* mutant backgrounds, we stained for Foxp1 (vLGE) and Sp8 (dLGE). Although the recovery of these two LGE compartments was limited in *Gsx2* null embryos at this stage (compare Fig. 5K with I), in *Gsx2^Q252R/EGFP^* embryos, their development remained intermediate (compare Fig. 5J with I and K, quantified in L), which correlates well with the upregulation/expansion of Gsx1 and the existence of the mutant Gsx2^Q252R^ protein, largely in the dorsal LGE regions.

### Striatal defects in *Gsx2^Q252R/EGFP^* mice

**Fig. 6.**
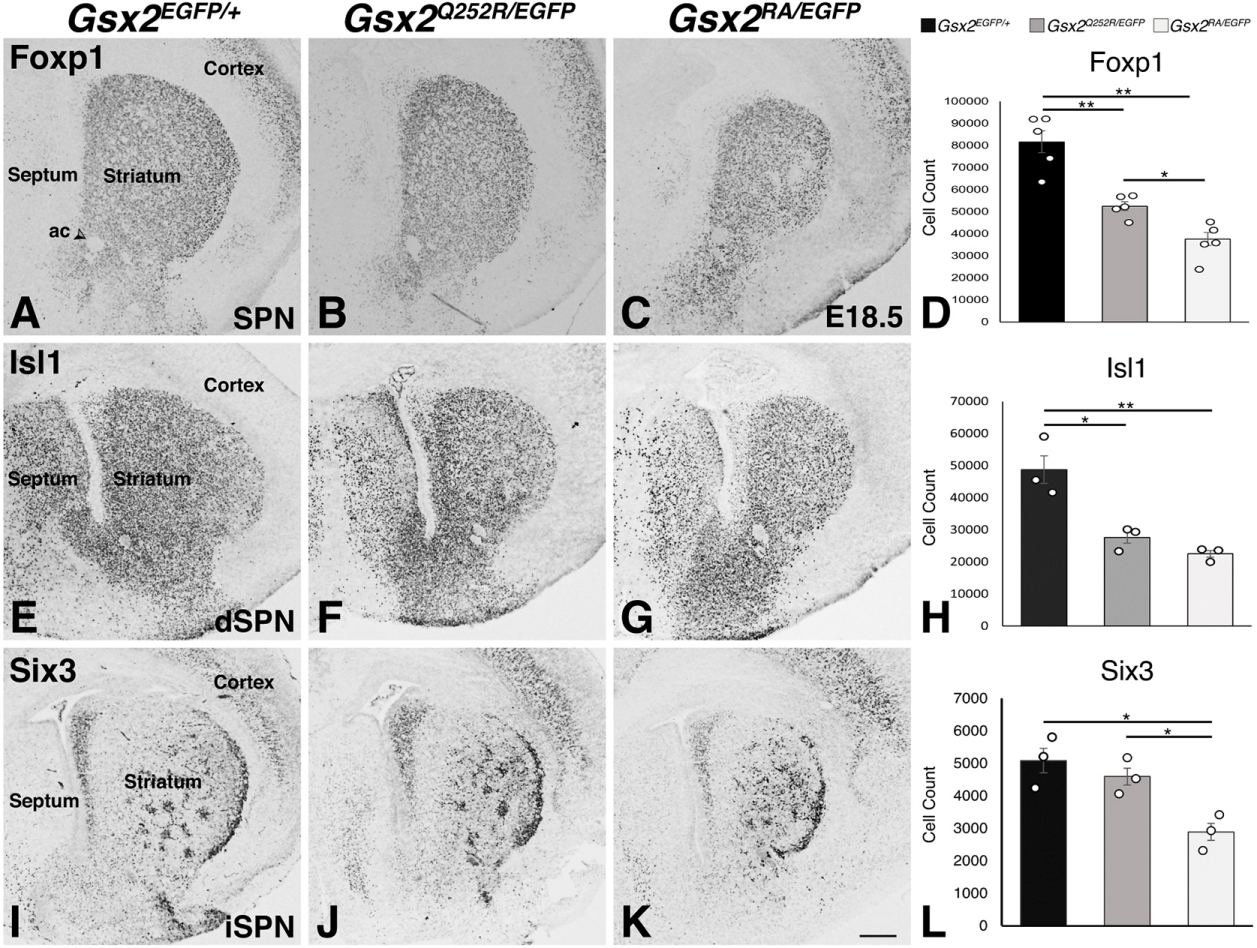
**Striatal projection neuron defects in *Gsx2^Q252R/EGFP^* and *Gsx2* null embryos at E18.5.** (A-L) Immunohistochemistry and quantifications for Foxp1 to analyse total striatal projection neurons (SPNs) (A-D), Isl1 to analyse direct pathway SPNs (dSPNs) (E-H) and Six3 to analyse indirect pathway SPNs (iSPNs) (I-L). A significant decrease in Foxp1^+^ cell count (81,645.6±4977.2 cells in controls) is observed with loss of *Gsx2* (37,601.3±2930.7 cells) (C,D) and, to an intermediate extent, with the Q252R mutation (52,507.4±1952.2 cells) (B,D). *Gsx2* null (22,511.7±1019.7 cells) (G) and Gsx2^Q252R/EGFP^ (27,633.7±1758.8 cells) (F) striatum both show a large reduction in Isl1 expression compared to that in WT (48,758±4313.8 cells) (E,H). Notably, although Six3 expression is severely decreased with loss of *Gsx2* (2889.7±260.5 cells) (K,L), no significant difference in cell count was observed between control striatum (5085.7±375.1 cells) (I) and *Gsx2^Q252R/EGFP^* striatum (4592.3±262.8 cells) (J,L). (D,H,L) Bar graphs show average cell count±s.e.m. Statistical analysis was performed using one-way ANOVA with Tukey post hoc (*n*=3, *P<0.05, **P<0.01). Scale bar: 200 µm. ac, anterior commissure (see arrowhead).

All three pathological *GSX2* variants in patients reported thus far exhibit severe basal ganglia dysgenesis despite one being a nonsense null truncation and the other two being missense variants within the HD (De Mori et al., 2019; Ürel-Demir et al., 2024). Given the intermediate phenotype observed in the *Gsx2^Q252R/EGFP^* LGE, we compared striatal development at E18.5 because *Gsx2* null animals are not viable after birth (Szucsik et al., 1997). Importantly, in rodents, more than 90% of the SPNs and striatal interneurons are generated by birth (Bayer, 1984); thus, E18.5 represents a good time point to examine the nascent striatal complex. Foxp1 marks all SPNs (both dSPNs and iSPNs) (Precious et al., 2016), and we found that E18.5 *Gsx2* null embryos exhibited a 54% reduction in Foxp1^+^ striatal cells (Fig. 6C) compared to those in controls (Fig. 6A,D). Comparatively, *Gsx2^Q252R/EGFP^* embryos displayed a 36% reduction in Foxp1^+^ striatal cells (Fig. 6B) compared to those in controls (Fig. 6A,D). Hence, the number of Foxp1^+^ cells in the *Gsx2^Q252R/EGFP^* embryonic striatum was significantly different from that observed in controls and nulls (Fig. 6D).

To examine dSPNs and iSPNs at E18.5, we quantified Isl1^+^ and Six3^+^ striatal cells, respectively. *Gsx2* nulls showed a 54% decrease in Isl1^+^ cell numbers (Fig. 6G,H), and a 43% decrease in Six3^+^ cell counts (Fig. 6K,L), compared to those of controls (Fig. 6E,I). In *Gsx2^Q252R/EGFP^* embryos, we measured a 43% reduction in Isl1^+^ cells (Fig. 6F,H) compared to those of controls, but no significant difference from those of *Gsx2* nulls (Fig. 6H). Interestingly, no significant change in the Six3^+^ cell count was noted between the *Gsx2^Q252R/EGFP^* striatum (Fig. 6J,L) and control striatum (Fig. 6I). However, the number of Six3^+^ cells in the *Gsx2^Q252R/EGFP^* striatum was significantly greater than that in the null striatum (Fig. 6J-L). This difference in the ratio of iSPNs to dSPNs in the *Gsx2^Q252R/EGFP^* striatum suggests that the mutant Gsx2^Q252R^ protein contributes differently to the specification of these SPN subtypes.

### Hindbrain defects and postnatal viability in *Gsx2^Q252R/EGFP^* and *Gsx2^Q252R/Q252R^* mice

**Fig. 7.**
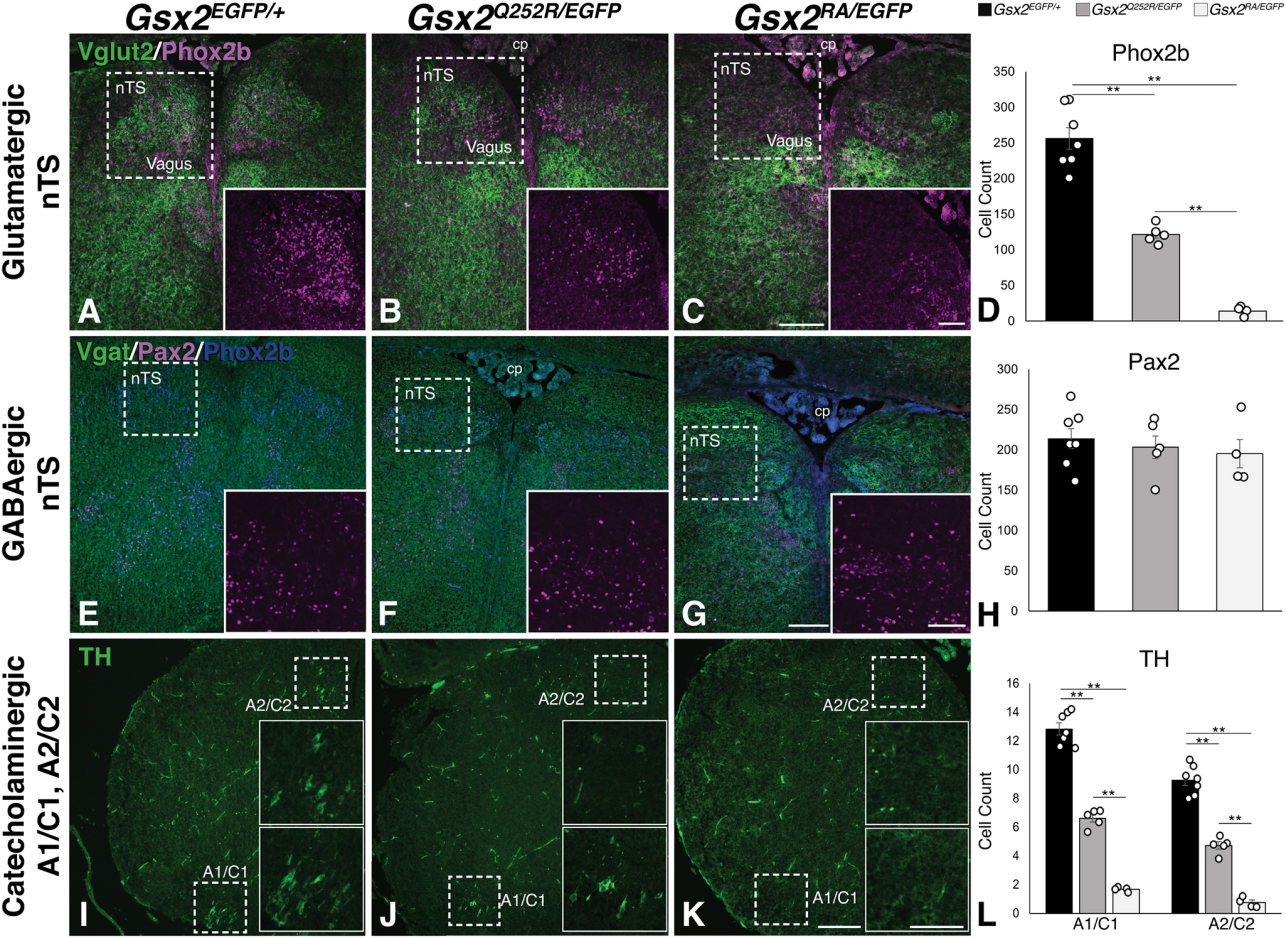
**E18.5 *Gsx2^Q252R/EGFP^* embryos show intermediate loss of glutamatergic nucleus tractus solitarius (nTS) neurons and catecholaminergic neurons in the A1/C1 and A2/C2 regions, both of which are nearly absent populations in *Gsx2* null embryos at birth**. (A-L) Coronal sections through the medulla of E18.5 control (A,E,I), *Gsx2^Q252R/EGFP^* (B,F,J) and *Gsx2^RA/EGFP^* (C,G,K) embryos and quantifications of cell counts (D,H,L). (A-C) Glutamatergic neurons of the nTS, marked with Vglut2 and Phox2b (256.4±15.1 cells in controls), are nearly lost in *Gsx2* null hindbrain (14.2±2.8 cells) (C) and decreased by ∼53% in *Gsx2^Q252R/EGFP^* hindbrain (121.5±5 cells) (B) (quantification shown in D). (E-G) GABAergic nTS Vgat^+^/Pax2^+^ (213.9±12.5 cells in controls) are preserved in *Gsx2^Q252R/EGFP^* embryos (203.4±14 cells) (F) and in *Gsx2* null embryos (195.3±17.6 cells) (G) (quantified in H). (I-K) Catecholaminergic neurons (TH^+^) in the nearby A1/C1 and A2/C2 regions (12.8±0.4 cells and 9.3±0.4 cells, respectively) are almost absent in *Gsx2* null hindbrain (1.7±0.1 and 0.8±0.2 cells, respectively) (K) but are decreased by ∼50% in *Gsx2^Q252R/EGFP^* hindbrain (6.6±0.3 and 4.7±0.3 cells) (J) (quantified in L). Bar graphs show average cell counts±s.e.m. Significance in quantifications were determined by one-way ANOVA using Tukey post hoc (*n*=4-7 for each genotype, *P<0.05, **P<0.01). Scale bars: 200 µm. cp, choroid plexus.

As mentioned above, *Gsx2* null animals become hypoxic, turn cyanotic and cease to breathe within 24 h of birth (Szucsik et al., 1997). In addition to the basal ganglia phenotypes in *Gsx2* null animals (Corbin et al., 2000; Toresson et al., 2000; Toresson and Campbell, 2001; Yun et al., 2003, 2001), these mutants exhibit defects in the nTS of the dorsocaudal hindbrain; however, no details were provided regarding the impact of the loss of Gsx2 function on distinct neuronal subtypes in this nucleus (Szucsik et al., 1997). The glutamatergic nTS neurons derive from the dA3 progenitor domain in the embryonic hindbrain, which is purported to express Gsx2, whereas the GABAergic nTS neurons arise from progenitors in the more ventral dB1 domain (Diek et al., 2022; Hernandez-Miranda et al., 2017; Storm et al., 2009). This nucleus incorporates barosensory and chemosensory information from the vagus and glossopharyngeal nerves to modulate respiratory responses to hypoxia and hypercarbia (Zoccal et al., 2014). It was, therefore, logically assumed that aberrant development of the nTS contributed to respiratory distress and premature death in these mice (Szucsik et al., 1997). Despite the hypomorphic basal ganglia phenotypes observed in *Gsx2^Q252R/EGFP^* and *Gsx2^Q252R/Q252R^* embryos, we found in this current study that newborn pups with these genotypes breathe and are fully viable. Thus, we compared the neuronal composition of the nTS prior to birth in E18.5 *Gsx2^EGFP/+^* (control), *Gsx2^EGFP/RA^* (null) and *Gsx2^Q252R/EGFP^* embryos. Glutamatergic (i.e. excitatory) nTS neurons express the vesicular glutamate transporter 2 (Vglut2; also known as Slc17a6) and the HD TF Phox2b, while the GABAergic nTS neurons express the vesicular GABA transporter (Vgat; also known as Slc32a1) and the HD TF Pax2 (Cheng et al., 2004; Diek et al., 2022; Hernandez-Miranda et al., 2018; Storm et al., 2009; Xia et al., 2022). Compared to controls, *Gsx2* nulls displayed almost complete loss of glutamatergic Phox2b neurons in the nTS (94% reduced) (Fig. 7A,C,D). Conversely, we found no change in the number of Pax2^+^ (i.e. GABAergic) neurons in the nTS of *Gsx2* nulls compared to that in controls (Fig. 7E,G,H). In comparison, *Gsx2^Q252R/EGFP^* embryos again displayed an intermediate phenotype, losing ∼53% of glutamatergic Phox2b^+^ neurons in the nTS (Fig. 7B,D). Similar to the *Gsx2* null embryos, no alteration in the number of Pax2^+^ (GABAergic) neurons was observed in the *Gsx2^Q252R/EGFP^* nTS (Fig. 7F,H). These data demonstrate that although glutamatergic nTS neurons are lost in *Gsx2* null embryos, one copy of the *Gsx2^Q252R^* allele is sufficient for roughly half of this population to form.

The nTS also possesses two developmentally related (i.e. dA3-derived) catecholaminergic (i.e. adrenergic and noradrenergic) neuronal groups: the dorsally positioned A2/C2 group and the ventrolaterally located A1/C1 groups, which integrate chemosensory information from the lungs to regulate breathing (Diek et al., 2022; Guyenet et al., 2013; Hernandez-Miranda et al., 2017; Malheiros-Lima et al., 2020; Pattyn et al., 1999; Qian et al., 2001; Roux et al., 2003; Rukhadze et al., 2017). These neurons can be visualized by tyrosine hydroxylase (TH) staining. However, they are also glutamatergic and express Phox2b (Malheiros-Lima et al., 2020; Pattyn et al., 1999; Qian et al., 2001; Roux et al., 2003). We assessed the status of TH^+^ neurons in the A1/C1 and A2/C2 groups of the different *Gsx2* mutants. Notably, *Gsx2* null embryos lacked nearly all catecholaminergic (i.e. TH^+^) neurons in both the A1/C1 (87% reduced) and A2/C2 regions (92% reduced), compared to those in controls (Fig. 7I,K,L), whereas *Gsx2^Q252R/EGFP^* embryos displayed a much less severe loss of TH^+^ neurons within the A1/C1 (48% reduced) and A2/C2 (49% reduced) regions (Fig. 7I,J,L). Therefore, one *Gsx2^Q252R^* allele is sufficient for the relative sparing of glutamatergic nTS neurons and the associated A1/C1 and A2/C2 catecholamine groups, compared to *Gsx2* nulls, suggesting that distinct thresholds of these neuronal subtypes underlie the breathing function and, ultimately, the postnatal viability of *Gsx2^Q252R/EGFP^* and *Gsx2^Q252R/Q252R^* mice.

## DISCUSSION

Until recently, no pathological human variants in *GSX2* had been reported. De Mori et al. (2019) were the first to report GSX2 variants associated with basal ganglia dysgenesis: one a presumptive null with a stop codon after amino acid 9 (p.S9*) and another a missense variant resulting in a p.Q251R change in the GSX2 HD. Both patients showed severe basal ganglia dysgenesis on MRI but were reported to survive at least until 5 and 14 years of age, respectively. Conversely, Ürel-Demir et al. (2024) recently reported a patient with a homozygous missense variant that led to p.W249C change in the HD and severe basal ganglia dysgenesis on MRI. This patient died from respiratory failure at 2 years of age and had an older sibling who died from respiratory failure at 4 months of age. Although this sibling was not sequenced, MRI imaging showed similar basal ganglia dysgenesis to that of the younger sibling (Ürel-Demir et al., 2024). Given that only one to two human patients have been found per variant, utilization of mouse genetics to model such variants allows a more complete and rigorous analysis of their mechanisms of pathogenesis.

**Fig. 8.**
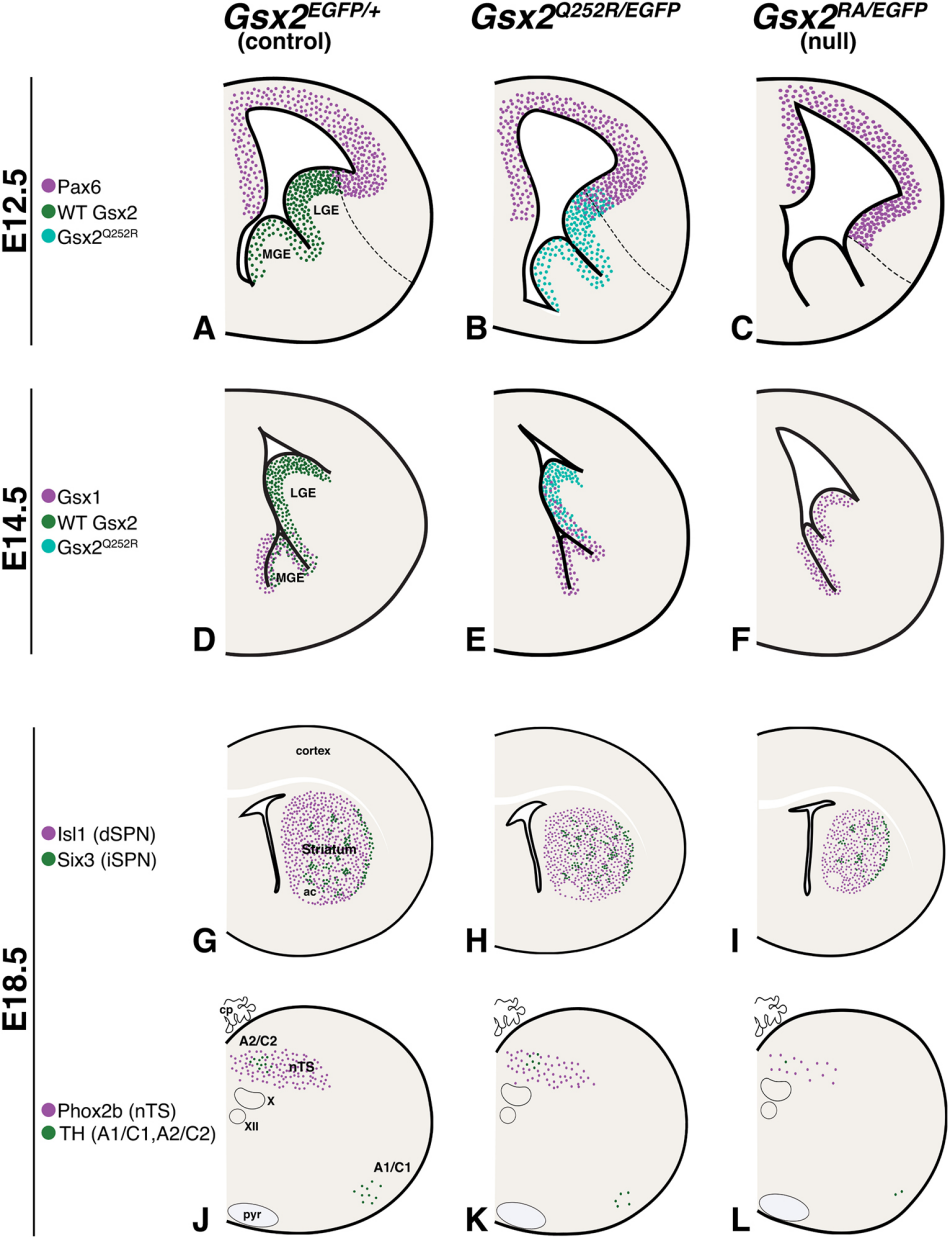
**Summary of alterations in *Gsx2^Q252R/EGFP^* and *Gsx2* null embryos throughout development.** (A-C) At E12.5, Gsx2 expression is truncated in *Gsx2^Q252R/EGFP^* embryos and absent in null embryos, leading to a proportional ventral expansion of the dorsal telencephalic regulator Pax6. (D-F) At E14.5, Gsx1 expression is scarce in the LGE of control embryos (D) and robustly upregulated within the LGE of *Gsx2* null embryos (F). Intermediate upregulation of Gsx1 is also present in the *Gsx2^Q252R/EGFP^* LGE at this stage, intermingled in the dorsal portion with Gsx2^Q252R^ (i.e. mutant)-expressing cells (E). (G-I) At E18.5, the striatal size of *Gsx2^Q252R/EGFP^* embryos is moderately reduced compared to that of control (G), with a relative sparing of iSPNs (i.e. Six3-expressing cells) versus dSPNs (i.e. Isl1-expressing cells) (H). *Gsx2* null embryos show a more severe reduction in striatal size and no preferential loss between dSPNs and iSPNs (I). (J-L) Significantly fewer glutamatergic nTS and catecholaminergic A1/C1 and A2/C2 neurons are present in the *Gsx2* null hindbrain (L) compared to control hindbrain (J). The *Gsx2^Q252R/EGFP^* hindbrain shows intermediate reduction in both of these neuronal subtypes (K), perhaps indicating a threshold for viability. ac, anterior commissure; cp, choroid plexus; pyr, pyramidal tract; X, dorsal motor nucleus of the vagus nerve; XII, hypoglossal nucleus.

In this study, we modelled the *GSX2^Q251R^* variant (De Mori et al., 2019) in mice by characterizing the homologous *Gsx2^Q252R^* mouse mutation. We observed a hypomorphic phenotype in these mice, as well as postnatal viability that is not observed in *Gsx2* nulls (Corbin et al., 2000; Szucsik et al., 1997; Toresson et al., 2000; Toresson and Campbell, 2001; Waclaw et al., 2009; Wang et al., 2009; Yun et al., 2003, 2001). Specifically, the *Gsx2^Q252R/EGFP^* and *Gsx2^Q252R/Q252R^* mutants showed an intermediate LGE phenotype (i.e. truncation and molecular misspecification), which is roughly half as severe as that observed in *Gsx2* nulls (schematized in Fig. 8A-C). Although it is unclear whether there is a difference in the severity of basal ganglia dysgenesis observed in *GSX2* null human patients versus those with the GSX2^Q251R^ variant (De Mori et al., 2019), the striatal (i.e. basal ganglia) dysgenesis observed at birth in animals expressing only the Gsx2^Q252R^ protein is reproducibly about half as severe as that observed in *Gsx2* nulls (schematized in Fig. 8G-I). Notably, the Gsx2^Q252R^ protein is localized to the nucleus both *in vitro* and *in vivo* and expressed in the correct spatial pattern during telencephalon development. Moreover, using a cell-based luciferase assay (Salomone et al., 2021), we found that the Gsx2^Q252R^ protein represses and activates gene expression via high-affinity binding sites *in vitro* similar to wild-type Gsx2. Gsx2 belongs to the Q50 HD TF subgroup that can also bind TAATGG sites with relatively high affinity (Berger et al., 2008; Bürglin and Affolter, 2016; Laughon, 1991). However, we found that the Gsx2^Q252R^ protein shows severely compromised binding *in vitro* to the Q50 DNA sequences (TAATGG), which represents as many as 277 of the wild-type Gsx2 DNA target sites in our previously published CUT&RUN data (Salomone et al., 2021). Therefore, reduced DNA binding likely underlies the observed hypomorphic function of the Gsx2^Q252R^ protein.

In this respect, Gsx2 normally represses *Pax6* in the LGE, and, in its absence, LGE progenitors upregulate Pax6, resulting in their misspecification to ventral pallial fates and, ultimately, the truncation of the LGE in *Gsx2* nulls (Toresson et al., 2000; Waclaw et al., 2010). Accordingly, we previously demonstrated that the LGE phenotype in *Gsx2* nulls can be partially ameliorated by inactivating *Pax6* (Toresson et al., 2000). Embryos expressing only the Gsx2^Q252R^ protein exhibit a less severe LGE truncation, and Pax6 misexpression is observed in VZ progenitors within the dorsal half of the LGE (Fig. 8A-C). These findings are consistent with the mutant Gsx2^Q252R^ protein failing to completely repress *Pax6* and thereby resulting in a more modest truncation of the LGE. It is unlikely that the dose of Gsx2^Q252R^ contributes to the phenotype in the *Gsx2^Q252R/EGFP^* LGE because the *Gsx2^Q252R/Q252R^* LGE showed nearly identical truncation and misspecification. Instead, the present findings suggest direct regulation of *Pax6* by Gsx2 because multiple Gsx2 CUT&RUN peaks were observed around the *Pax6* gene locus (Salomone et al., 2021), and we observed selectively reduced binding of the Gsx2^Q252R^ protein to a number of the footprinted regions that normally bind wild-type Gsx2. This further supports the notion that impaired binding of the Gsx2^Q252R^ protein on certain Gsx2 target sites underlies a significant portion of the observed phenotype in the *Gsx2^Q252R/Q252R^* and *Gsx2^Q252R/EGFP^* LGE.

Previous studies have shown that the limited striatal development observed in *Gsx2* nulls is critically dependent on its family member Gsx1 (Toresson and Campbell, 2001; Yun et al., 2003). In fact, Gsx1 upregulates throughout the *Gsx2* null LGE and partially compensates for the loss of Gsx2 function in striatal development within the mutant. Yet, even with Gsx1 upregulation, the *Gsx2* null striatum is greatly compromised. It seems unlikely that the upregulation of Gsx1 in *Gsx2* null LGEs is due directly to the loss of Gsx2 because it is delayed by at least 2 gestational days (Toresson and Campbell, 2001; Wang et al., 2009; Yun et al., 2003). However, direct regulation is possible because Gsx2 CUT&RUN peaks were observed around the *Gsx1* locus (Salomone et al., 2021). Our previous work suggested that Gsx2 maintains LGE progenitors in an undifferentiated state, whereas Gsx1 promotes neuronal differentiation (Pei et al., 2011). Interestingly, we found that Gsx2^Q252R^- and Gsx1-expressing progenitors are abnormally intermingled in the dorsal half of the *Gsx2^Q252R/EGFP^* LGE at E14.5, which is not the case for wild-type LGE progenitors. Therefore, it is likely that the Gsx2^Q252R^ protein is not efficient at maintaining LGE cells in a progenitor state, leading to the precocious generation of Gsx1-expressing progenitors undergoing differentiation. Nevertheless, the observed upregulation of Gsx1 in combination with a partially functional Gsx2 (i.e. Gsx2^Q252R^) likely plays an important role in generating a striatum in *Gsx2^Q252R/EGFP^* mice that is more developed than that in *Gsx2* nulls (schematized in Fig. 8D-F). As mentioned above, the MRI findings presented in the De Mori et al. (2019) study suggest that both the S9* null and Q251R HD variants result in similar basal ganglia dysgenesis, which is not the case in the *Gsx2^Q252R^* and *Gsx2* null mice presented here. Thus, it could be that, in humans, *GSX1* does not compensate for the loss/dysfunction of *GSX2*, resulting in similar basal ganglia dysgenesis between the two variants.

Overall, the *Gsx2^Q252R/EGFP^* phenotype resembles a less severe *Gsx2* null phenotype (i.e. hypomorph) for most observations. However, the balance between dSPNs and iSPNs marks a deviation from this pattern. At E18.5, there are ∼40-50% fewer dSPNs and iSPNs in *Gsx2* null than in control mice. However, in the *Gsx2^Q252R/EGFP^* perinatal striatum, the number of dSPNs was significantly reduced compared to that in controls, but iSPNs were relatively spared (Fig. 8G-I). This imbalanced striatal output could be substantial, considering that the two pathways are known to function primarily in an antagonistic manner to facilitate proper movement (Obeso et al., 2002). However, the severe dystonia reported in both the GSX2^S9*^ and *GSX2^Q251R^* patients (De Mori et al., 2019) could make it difficult to assess further neurological deficits between patients.

Although much of the *Gsx2^Q252R^* phenotype is similar to, but less severe than, the *Gsx2* null phenotype, the apparent imbalance of iSPNs to dSPNs in the *Gsx2^Q252R^* mutant striatum is not observed in *Gsx2* null striatum*.* This suggests that either the key target genes for iSPN specification can be properly regulated by the Gsx2^Q252R^ protein or that this mutant HD protein has aberrant function(s) in the generation of this SPN subtype. For example, the selective DNA binding (e.g. TAATTA but not TAATGG) of Gsx2^Q252R^ could allow for and/or explicitly promote iSPN specification. Alternatively, the Q252R mutation could ectopically bind an alternative subset of DNA sequences. In fact, HD TFs with a basic K residue at position 50 (e.g. Otx2) (Berger et al., 2008; Bürglin and Affolter, 2016; Laughon, 1991), which is similar to the R found in the Gsx2^Q252R^ variant, have been shown to have distinct DNA binding preferences from Q50 HDs. This is consistent with past bacterial hybrid specificity screens as R50s HDs were found to prefer TAAN(A/T)C sequences (Chu et al., 2012). Aberrant binding of Gsx2^Q252R^ to K50 sequences *in vivo* could lead to misregulation of genes not usually within the Gsx2 gene regulatory network, hence misspecifying SPNs and resulting in the observed altered ratio of iSPNs to dSPNs at birth.

The most notable difference in phenotypes between the *Gsx2^Q252R^* and *Gsx2* null mutants is that the former survive postnatally. The recent report from Ürel-Demir et al. (2024) describing respiratory failure in a patient with a homozygous variant in *GSX2* is consistent with the respiratory failure observed in *Gsx2* null mice. Szucsik et al. (1997) suggested that neuronal defects in the nTS of the caudal hindbrain underlie the respiratory failure in newborn *Gsx2* nulls. However, no detailed analysis was performed to assess the nTS neuronal defects that occur in these mutant mice. Our data demonstrate that *Gsx2* is required for the formation of glutamatergic nTS neurons and their associated A1/C1 and A2/C2 catecholaminergic neurons (schematized in Fig. 8J,L). These neurons comprise the dorsal respiratory group and are required for inspiration and regulation of breathing rhythms (Diek et al., 2022; Fu et al., 2019; Guyenet et al., 2013; Malheiros-Lima et al., 2020; Pattyn et al., 1999; Qian et al., 2001; Roux et al., 2003; Zoccal et al., 2014). In keeping with the observed hypomorphic basal ganglia phenotypes, perinatal *Gsx2^Q252R/EGFP^* mutants exhibit a 53% reduction in glutamatergic nTS neurons and a similar reduction in A1/C1 (48%) and A2/C2 (49%) catecholaminergic neurons (schematized in Fig. 8J-L). Given that *Gsx2^Q252R/EGFP^* and *Gsx2^Q252R/Q252R^* animals breathe and thus survive, it seems that at least this threshold of glutamatergic nTS neurons and/or A1/C1 and A2/C2 catecholaminergic neurons is required for normal modulation of respiratory function and postnatal survival. Interestingly, a recent report showed a correlation between bilateral nTS lesions and respiratory failure at a young age (Parayil Sankaran et al., 2022). Moreover, previous studies have shown alterations in catecholamine neurons in the A1/C1 and A2/C2 regions of infants who died from sudden infant death syndrome (SIDS) (Denoroy et al., 1987; Obonai et al., 1998). Polymorphisms in the *TH* gene have also been clearly associated with SIDS (Courts and Madea, 2011; Klintschar et al., 2008).

In conclusion, by modelling the *GSX2^Q251R^* variant (De Mori et al., 2019) in mice (i.e. *Gsx2^Q252R^*), we have demonstrated a hypomorphic phenotype compared to that of *Gsx2* nulls. The hypomorphic function is likely due to the selectively reduced/loss of binding to a significant portion of wild-type Gsx2 DNA binding targets. Finally, the major difference observed between *Gsx2^Q252R^* and *Gsx2* null mutants is the postnatal viability of the former. Our hindbrain analysis points to a critical threshold of glutamatergic nTS and/or A1/C1 and A2/C2 catecholaminergic neurons for correct breathing control and thus viability.

## MATERIALS AND METHODS

### Cloning and cell transfections

Wild-type *Gsx2* complementary DNA (cDNA) was cloned into pCDNA6 vector using EcoRV and XhoI enzymes, as previously described (Roychoudhury et al., 2020). The *Gsx2^Q252R^* construct was generated using site-directed mutagenesis in a PM11 vector, using the following primers and restriction enzyme (Nde1, Xho1) sites: Gsx2_NdeI_167 (1234js), 5′-GTACATATGCCGCAGCACCACGCACCTGTC-3′; Gsx2_Q252R_rev (1019bc), 5′-GCTTCACGCGACGGTTCCGAAACCAGATTTTCACC-3′; Gsx2_XhoI_305 (1163js), 5′-GTACTCGAGTTACAAGGGGGAAATCTCCTTGTC-3′; and Gsx2_Q252R_fwd (1018bc), 5′-GGTGAAAATCTGGTTTCGGAACCGTCGCGTGAAGC-3′. The Q252R fragment was then transferred from PM11 to pCDNA6 using Xho1 and Kpn1 restriction enzymes, and the resulting Gsx2 Q252R pCDNA6 construct was sequence confirmed. HEK293T cells (RIKEN Bioresource Research Center, Kyoto, Japan) were plated at 0.5×10^6^ cells/well in a six-well plate and transfected 24 h later using Lipofectamine 3000 (Invitrogen, L3000008) as described by the manufacturer's protocol. Medium was changed 24 h later, and cells were fixed ∼48 h after transfection. Following fixation, cells were stained for Gsx2 using immunocytochemistry, and nuclei were counterstained with 4′,6-diamidino-2-phenylindole (DAPI), as described below.

### EMSAs

Gsx2^167-305^ proteins were purified from bacteria as previously described (Uhl et al., 2010). Protein purity was determined via SDS-PAGE and Coomassie Blue staining (Fig. S1). Protein concentrations were determined via Bradford assay. EMSAs were performed as previously described (Cain et al., 2023). Probe sequences were as follows: consensus Gsx2 binding site probe, 5′-CGGGCTAATTAGGCCTAGTGCGGGCGTGGCT-3′; Q50 probe, 5′-CGGGCTAATGGGGCCTAGTGCGGGCGTGGCT-3′. Fig. 4 probes were as follows: PAX6_M1, 5′-CATTGATTTATTAGGCTAGTAGTGCGGGCGTGGCT-3′; PAX6_M2, 5′-TGGAGGATGATGACAGAGGTAGTGCGGGCGTGGCT-3′; PAX6_M3, 5′-CTGAGACAAATGAGCACTGTAGTGCGGGCGTGGCT-3′; PAX6_M4, 5′-AGCTGTGTAATTAAATTTCTAGTGCGGGCGTGGCT-3′; PAX6_M5, 5′-CAGTGTTAAATAAGGGAGGTAGTGCGGGCGTGGCT-3′; PAX6_M6, 5′-CTTTGGTTAAAAAGGTGATTAGTGCGGGCGTGGCT-3′; and PAX6_M7, 5′-AGGAAGCTCATTAAAAATGTAGTGCGGGCGTGGCT-3′. Probes were annealed as previously described (Roychoudhury et al., 2020). Binding reactions containing 34 nM of the indicated DNA probes and 100, 200 and 400 nM of purified Gsx2 and Gsx2^Q252R^ proteins were mixed and incubated in the dark at room temperature for 20 min prior to gel electrophoresis. EMSAs were imaged via a Li-Cor Odyssey CLx scanner.

### ITC

ITC experiments were performed as described in Webb et al. (2024) using a Microcal VP-ITC microcalorimeter. All samples were dialyzed overnight to ensure buffer match (the buffer contained 50 mM sodium phosphate, pH 6.5 and 150 mM NaCl). DNA duplexes (see Table S1 for probe sequences and reaction conditions) were placed in the syringe at ∼100 μM, and purified wild-type Gsx2 or Gsx2^Q252R^ proteins were placed in the cell at ∼10 μM at 20°C. For titrations, an initial 1 μl injection was followed by 19 injections of 14 μl. Experiments were conducted in triplicate, and analysis was performed using ORIGIN and fit to a one-site binding model using equations from the ITC Data Analysis in Origin Tutorial Guide.

### Luciferase assays

Luciferase assays were performed in mouse mK4 cells to test the transcriptional activity of wild-type Gsx2 versus Gsx2^Q252R^ proteins on ‘M’ versus ‘D’ Gsx2-binding sites, as previously described (Salomone et al., 2021). The pCDNA6 constructs containing either Gsx2 or Gsx2^Q252R^ cDNAs were used for the luciferase assays, which were performed in triplicate. Cells were harvested 48 h after transfection and lysed, and luciferase activity was analysed using a Promega dual-luciferase assay kit and GloMax luminometer. To control for transfection efficiency, all firefly luciferase values were normalized to Renilla luciferase. Bar graphs show average relative luciferase values±s.e.m. with Gal4-VP16 alone set to 100.

### Animals

All mouse work and protocols were compliant with Cincinnati Children's Hospital Medical Center Institutional Animal Care and Use Committee and the National Institutes of Health. Mice were maintained on an outbred CD1 background. The following previously generated mouse lines were used and genotyped as described: *Gsx2^EGFP^* (Wang et al., 2009) and *Gsx2^RA^* (Waclaw et al., 2009).

The *Gsx2^Q252R^* allele was generated by the Transgenic Animal and Genome Editing core at Cincinnati Children's Hospital Medical Center using a CRISPR-Cas9 and donor oligonucleotide approach. The single-guide RNA (sgRNA) CCTTCTTGTGCTTCACGCGACGG was delivered to embryos using electroporation, along with a Q252R knock-in donor oligonucleotide sequence (TATGTACCTGTCCCGACTCCGGAGAATCGAGATCGCGACATACCTAAACCTGTCAGAGAAGCAGGTGAAAATCTGGTTCCGGAATCGACGGGTGAAGCACAAGAAGGAGGGGAAAGGCGCTTCGA) and a wild-type donor sequence (TATGTACCTGTCCCGACTCCGGAGAATCGAGATCGCGACATACCTAAACCTGTCAGAGAAGCAGGTGAAAATCTGGTTCCAGAATCGACGTGTGAAGCACAAGAAGGAGGGGAAAGGCGCTTCGA) to decrease the likelihood of lethality due to homozygous knock-in mutations, as seen in *Gsx2* null mice. The knock-in donor sequence replaces CAG with CGG coding for arginine (R) instead of glutamine (Q) at amino acid position 252 of the protein, as well as additional silent mutations creating a Bspe1 digest site allowing for detection of the mutant allele. Genotyping primers F-Gsx2_Q252R (5′-GAGCTGGAGCGAGAATTCTCTTC-3′) and R-Gsx2_Q252R (5′-CTAGAACTTTGGTCCCTGTGCTG-3′) amplify both wild-type and Q252R DNA to generate a 348 bp PCR product, which was then digested with BspE1. DNA carrying the Q252R mutation digests to 46/60/242 bp fragments (see Fig. 2). Our CRISPR experiment generated two founders with the desired mutation that were confirmed by Sanger sequencing. Both founder lines had a consistent forebrain phenotype upon preliminary characterization, and the line that was used was selected based on most successful breeding.

### Tissue preparation

For embryonic analysis, detection of a vaginal plug was noted as day E0.5, and embryos were harvested at the selected timepoints. Embryos (E12.5 and E14.5) or heads (E18.5) were fixed overnight in 4% paraformaldehyde, then washed in PBS. E18.5 brains were dissected out of heads prior to sectioning; brains from earlier timepoints were left in the head for sectioning. Samples were placed in 20% sucrose for cryopreservation, then sectioned at a thickness of 12 µm on a cryostat and collected on (+) charged slides (Fisher Scientific).

### Immunohistochemistry

Cryosections were stained on slides using a humid chamber and with solutions prepared using a potassium phosphate-buffered saline (KPBS) solution with 0.01% Triton X-100. For immunofluorescence, primary antibodies were incubated overnight at room temperature, followed by incubation with a secondary antibody conjugated to a fluorophore for 2 h, with three sets of 10 min washes between incubations. Slides were coverslipped using DAPI Fluoromount (Southern Biotech, 0100-20). Diaminobenzidine (DAB) immunohistochemical staining was conducted using the same primary antibody incubation followed by a 2 h incubation with a secondary antibody conjugated to biotin and a subsequent 1 h incubation with ABC solution (Vector Laboratories, PK6100). The stain was developed using DAB as a chromogen. Once developed, slides were dried overnight before ethanol dehydration and coverslipping with DPX mounting medium (Sigma-Aldrich, 06522). Gsx1 staining was performed with amplification with tyramide 488 (Biotium, CF488A) as described in the manufacturer's protocol. To reduce background of certain immunofluorescence stains, TrueBlack (Biotium, 23007) was used prior to adding coverslips as described in the manufacturer's protocol.

Primary antibodies were used at the following dilutions: guinea pig anti-Ascl1 (1:10,000; Kim et al., 2008; a gift from J. Johnson, UT Southwestern, Dallas, TX, USA), rat anti-BrdU (1:200; Serotec, MCA2060), rabbit anti-Dlx2 (1:500; Lindtner et al., 2019; a gift from J. Rubenstein, UCSF, San Francisco, CA, USA), rabbit anti-Foxp1 (1:4000; Abcam, ab16645, RRID:AB_732428), guinea pig anti-Gsx1 (1:20,000 with tyramide-488; Qin et al., 2017), rabbit anti-Gsx2 (1:2000-3000; Toresson et al., 2000), chicken anti-EGFP (1:1000; Aves, GFP-1010, RRID:AB_2307313), goat anti-Isl1 (1:500; R&D Systems, AF1837, RRID:AB_2126324), rabbit anti-ZFP503 (Nolz1) (1:1000; Sigma-Aldrich, HPA026848, RRID:AB_10610704), sheep anti-Pax6 (1:500; R&D Systems, AF8150, RRID:AB_2827378), rabbit anti-Pax2 (1:200; Covance, PRB-276P, RRID:AB_291611), goat anti-Phox2b (1:500; Santa Cruz Biotechnology, sc13226, RRID:AB_2163613), goat anti-Sp8 (1:5000; Santa Cruz Biotechnology, sc104661, RRID:AB_2194626), rabbit anti-Six3 (1:1500; Rockland, 600-401-A26, RRID:AB_11180063), chicken anti-TH (1:500; Aves, TYH, RRID:AB_10013440), guinea pig anti-Vgat (1:500; Synaptic Systems, 131004, RRID:AB_887873), guinea pig anti-Vglut2 (1:500; Millipore, AB2251-1) and rabbit anti-Ki67 (1:500; Novocastra, NCL-Ki67p).

The following fluorescently labelled secondary antibodies from Jackson ImmunoResearch were used at 1:500 dilution: anti-rabbit Alexa Fluor 488 (711-546-152, RRID:AB_2340619), Alexa Fluor 594 (711-586-152, RRID:AB_2340622) and Alexa Fluor 647 (711-606-152, RRID:AB_2340625); anti-guinea pig Alexa Fluor 647 (706-606-148, RRID:AB_2340477); anti-goat Alexa Fluor 594 (705-586-147, RRID:AB_2340434) and Alexa Fluor 647 (705-606-147, RRID:AB_2340438); anti-chicken Alexa Fluor 488 (703-546-155, RRID:AB_2340376); and anti-rat Alexa Fluor 488 (712-546-150, RRID:AB_2340685). Biotinylated secondary antibodies from Jackson ImmunoResearch were used at 1:500 dilution: anti-goat (705-066-147, RRID:AB_2340398), anti-guinea pig (706-066-148, RRID:AB_2340452) and anti-rabbit (711-066-152, RRID:AB_2340594). Anti-sheep Alexa Fluor 568 fluorescent secondary antibody was purchased from Invitrogen (A21099, RRID: AB_10055702) and used at 1:500 dilution.

### BrdU labelling

Pregnant females were injected with BrdU (100 mg/kg) at E12.5, and embryos were collected 1 h post-injection, fixed and genotyped as above. Tissue was processed and sectioned as described. Sections were then treated with 2 N HCl for 50 min at room temperature for antigen retrieval and subjected to immunofluorescence as described above.

### Imaging and statistical analysis

Images were captured on a Ti-2 Eclipse widefield microscope (Nikon) using Nikon Elements AR software or an BX51 microscope equipped with epifluorescence (Olympus). Adobe Photoshop was used to optimize brightness and pseudocolour images. To quantify the LGE truncation in *Gsx2^Q252R/Q252R^*, *Gsx2^Q252R/EGFP^* and *Gsx2^EGFP/RA^* mice at E12.5, and *Gsx2^Q252R/EGFP^* and *Gsx2^EGFP/RA^* mice at E14.5, the expression domains of Gsx2 or EGFP were used to trace the circumference of the LGE ventricular surface (dotted white lines in Figs 2 and 3) from the ventralmost point of the MGE/LGE sulcus to the dorsolateral edge of Gsx2 or GFP expression within the LGE. The length of this ‘LGE arc’ was measured on each E12.5 section containing GFP or Gsx2 staining, and all measurements for one brain were added together. The reported arc length was the average total of three brains from each genotype and represented as single dots in the bar graphs.

Cell counts were determined for the following stains: BrdU, Ascl1, Dlx2, Sp8, Nolz1, Six3, Isl1 and Ki67 at E12.5; Gsx1, Gsx2, Foxp1 and Sp8 at E14.5; and Six3, Isl1 and Foxp1 at E18.5. These stains were imaged at 10× magnification and quantified using NIS Elements (Nikon) bright spot detection. Striatal cell counts of each brain section were added together to generate the cell count, and the total number of cells from three brains was averaged for the final count reported.

Hindbrain sections were imaged at 100× magnification, and NIS Elements bright spot detection was used to quantify numbers of Phox2b^+^ and Pax2^+^ nuclei in the nTS. The location and structure of the nTS was identified by anatomical landmarks, which were used to define the region of interest for cell counts. TH^+^ neurons were quantified by hand counting TH^+^/DAPI^+^ cells in each section. For hindbrain analysis, *n*=7 *Gsx2^GFP/+^*, *n*=4 *Gsx2^EGFP/RA^* and *n*=5 *Gsx2^Q252R/EGFP^* animals were quantified. The counts for each section were averaged per animal, and each data point on a graph represents one animal.

Statistical significance for quantifications was determined by one-way ANOVA with Tukey post hoc, using *P*<0.05 as the threshold for significance. Bar graphs shown indicate the average measurement±s.e.m., with each dot representing a replicate measurement.

## Supplementary Material

10.1242/dmm.052110_sup1 Supplementary information
