# Supplementary material for: Modelling a pathological GSX2 variant that selectively alters DNA binding reveals hypomorphic mouse brain defects
Source: Dis Model Mech. 2025 Feb 20;18(2):dmm052110. doi: 10.1242/dmm.052110 (PMC11876842; doi:10.1242/dmm.052110)
Supplement: Supplementary information [file dmm-18-052110-s1.pdf]

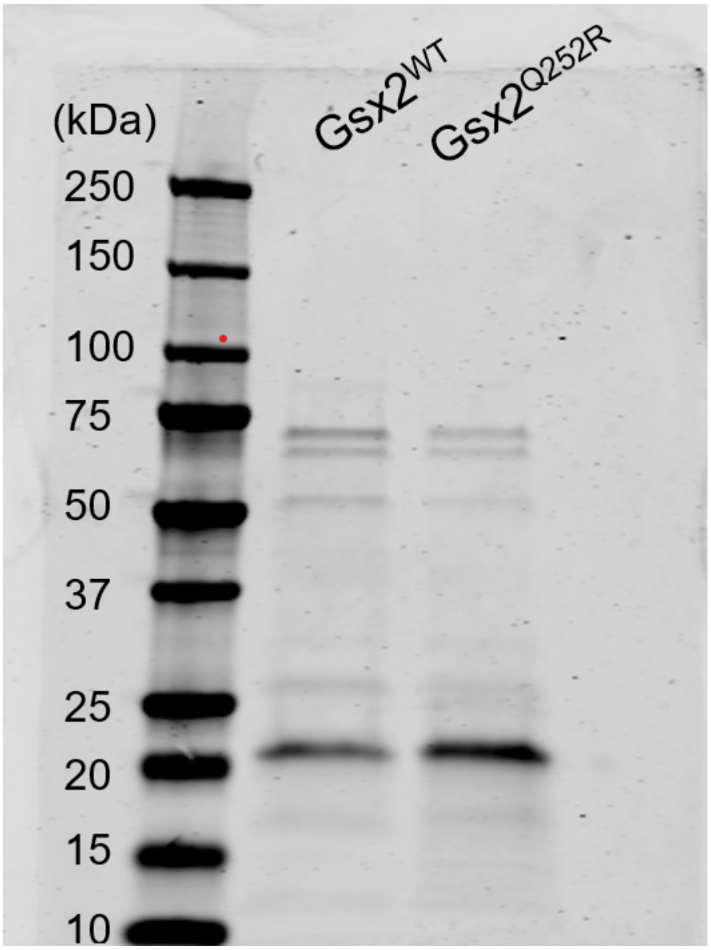

Fig. S1. Coomassie Blue.
SDS-PAGE used to assess purity of proteins used in EMSAs. 2.5 μM of protein was loaded into each lane.

Disease Models & Mechanisms • Supplementary information

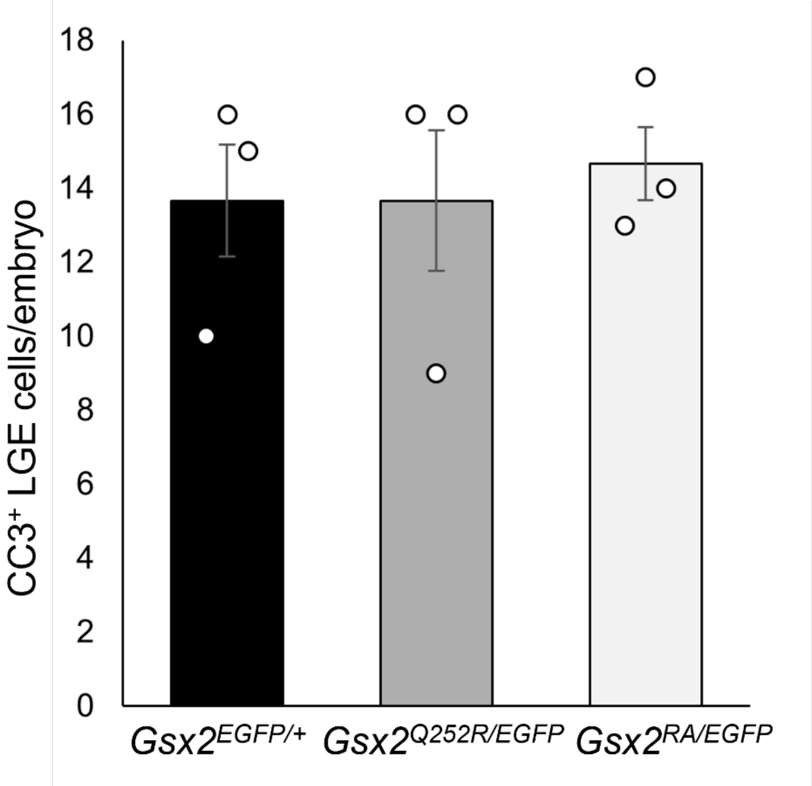

Fig. S2. Quantification of cleaved caspase-3 in the LGE of *Gsx2$^{EGFP/+}$*, *Gsx2$^{Q252R/EGFP}$* and *Gsx2$^{RA/EGFP}$* (i.e. null) embryos.

Cleaved caspase-3-positive ($CC3^+$) cells counted in the LGE of each embryo at E12.5. Bar graphs indicate mean ±s.e.m with dots showing individual embryos, *n*=3 for each genotype. No statistically significant difference was seen between the genotypes, as determined using a one-way ANOVA with Tukey post-hoc ($p=0.91$).

Disease Models & Mechanisms • Supplementary information

Table S1. Calorimetric data of Gsx2 and Gsx2^{Q252R} HD binding to DNA monomer sites.

Cell (Protein 10uM)	Syringe (DNA 100uM)	Temp (K)	N	K (M^{-1})	Kd (nM)	dG (kcal/Mol)	dH (kcal/Mol)	-TdS (kcal/Mol)
mGsx2 203-264	TGAGC**TAATTA**AAGC	293	1.17	$3.51x10^8$	3	-11.45	-5.12	-6.33
mGsx2 203-264 Q252R	TGAGC**TAATTA**AAGC	293	0.957	$9.34x10^7$	11	-10.69	-5.51	-5.19
mGsx2 203-264	TGAGC**TAATGG**AAGC	293	1.15	$3.14x10^7$	32	-10.06	-6.46	-3.60
mGsx2 203-264 Q252R	TGAGC**TAATGG**AAGC	293	-	-	-	-	-	-

The thermodynamic parameters of binding between the Gsx2 proteins and DNA probes are indicated in the above table. Constructs used are WT Gsx2 HD (noted as mGsx2 203-264) and Q252R HD (noted as mGsx2 203-264 Q252R). Homeodomain-binding sites are highlighted in bold.

Disease Models & Mechanisms • Supplementary information
